# Skipping of exon 10 in* Axl* pre-mRNA regulated by PTBP1 mediates invasion and metastasis process of liver cancer cells

**DOI:** 10.7150/thno.42010

**Published:** 2020-04-27

**Authors:** Lianghua Shen, Sijia Lei, Bin Zhang, Shuaiguang Li, Luyuan Huang, Alexander Czachor, Mason Breitzig, Yuman Gao, Meiyan Huang, Xuemei Mo, Qing Zheng, Hanxiao Sun, Feng Wang

**Affiliations:** 1 Institute of Genomic Medicine, College of Pharmacy, Jinan University, Guangzhou 510632, China.; 2 International Cooperative Laboratory of Traditional Chinese Medicine Modernization and Innovative Drug Development of Chinese Ministry of Education (MOE), College of pharmacy, Jinan University, Guangzhou 510632, China.; 3 Guangdong Provincial Key Laboratory of Stem Cell and Regenerative Medicine, Key Laboratory of Regenerative Biology, South China Institute for Stem Cell Biology and Regenerative Medicine, Guangzhou Institute of Biomedicine and Health, Chinese Academy of Sciences, Guangzhou 510530, China.; 4 Department of Internal Medicine, Morsani College of Medicine, University of South Florida, 12901 Bruce B. Downs Blvd, MDC 19, Tampa, FL 33612, USA.; 5 Brown School of Social Work, Washington University in St. Louis, St. Louis, MO, USA

**Keywords:** Axl, alternative splicing, PTBP1, tumor metastasis, liver cancer cells

## Abstract

The Axl gene is known to encode for a receptor tyrosine kinase involved in the metastasis process of cancer. In this study, we investigated the underlying molecular mechanism of Axl alternative splicing.

**Methods**: The expression levels of PTBP1 in hepatocellular carcinoma (HCC) tissues were obtained from TCGA samples and cell lines. The effect of *Axl-L*, *Axl-S*, and PTBP1 on cell growth, migration, invasion tumor formation, and metastasis of liver cancer cells were measured by cell proliferation, wound-healing, invasion, xenograft tumor formation, and metastasis. Interaction between PTBP1 and Axl was explored using cross-link immunoprecipitation, RNA pull-down assays and RNA immunoprecipitation assays.

**Results**: Knockdown of the PTBP1 and exon 10 skipping isoform of Axl (Axl-S), led to impaired invasion and metastasis in hepatoma cells. Immunoprecipitation results indicated that* Axl-S* protein binds more robustly with Gas6 ligand than *Axl-L* (exon 10 including) and is more capable of promoting phosphorylation of ERK and AKT proteins. Furthermore, cross-link immunoprecipitation and RNA-pulldown assays revealed that PTBP1 binds to the polypyrimidine sequence**(TCCTCTCTGTCCTTTCTTC)** on *Axl*-Intron 9. MS2-GFP-IP experiments demonstrated that PTBP1 competes with U2AF2 for binding to the aforementioned polypyrimidine sequence, thereby inhibiting alternative splicing and ultimately promoting *Axl-S* production.

**Conclusion**: Our results highlight the biological significance of *Axl-S* and *PTBP1* in tumor metastasis, and show that PTBP1 affects the invasion and metastasis of hepatoma cells by modulating the alternative splicing of *Axl* exon 10.

## Introduction

Liver cancer is a significant threat to the wellbeing of mankind and is one of the world's major health problems. According to data released by the International Agency for Research on Cancer in 2015, more than 8.8 million people die of cancer every year worldwide, of which about 720,000 are attributable to liver cancer 1. Furthermore, more than 90% of patients with liver cancer die as a result of metastasis 2. Therefore, studies of metastasis mechanisms have far-reaching significance for the treatment of liver cancer. More specifically, studies have shown that alternative splicing (AS) plays an important role in the development and metastasis of cancer 3-5. Taken together, defining the molecular mechanism of AS provides a new angle from which to study the mechanisms of liver cancer metastasis.

The Axl receptor, a member of the TAM (Tyro3, Axl, Mer) family of receptor tyrosine kinases, has attracted significant attention as a promising oncology target 6. Axl consists of three parts: (1) an extracellular domain, which binds to a ligand and is linked by two immunoglobulin (Ig) domains and two fibronectin type III (FNIII) repeats; (2) an intracellular region, which is a tyrosine kinase domain characterized by autophosphorylation; (3) and a transmembrane region. The two highly homologous ligands of TAMs are Growth Arrest-specific protein 6 (Gas6) and protein S 7. Gas6 has the highest affinity for Axl 8, while protein S binds primarily to Tyro3 and Mer 9. Gas6 binds to the immunoglobulin-like domain of the Axl extracellular domain, causing receptor dimerization with a 2:2 stoichiometric configuration of Gas6 and Axl 10. Next, intracellular tyrosine residues are phosphorylated and subsequent Gas6/Axl phosphorylation triggers multiple signaling pathways (e.g. PI3K-AKT, AKT/GSK3β/β-catenin, MAPK-ERK signaling pathway) mediating cell survival, proliferation, invasion, and metastasis 11-15.

Analysis of the NCBI database identified 20 exons in the human Axl gene. Of these 20, exon 10 is the only variable exon, the others are constitutive exons. Comparison of transcript sequences revealed that the AS mode between Axl-isoform 2 and Axl-isoform 1 is exon 10 skipping. Analysis of the Human Protein Reference database (http://hprd.org/summary?hprd_id=00171&isoform_id=00171_1&isoform_name=Isoform_1) identified that the insertion of the exon 10 fragment, whose amino acid sequence is located between the fibronectin type 3 (FN3) and transmembrane domains. Whether the deletion of these nine amino acids will lead to functional or structural differences between Axl-S and Axl-L. It has attracted great attention from us. The AS events of Axl exon 10 are widespread with multiple animal species (e.g. human, cow, dog, horse 16, and mouse). A study by Jong et al. showed that the results of 3D structural analysis indicated that horse Axl-S (isoform 2) of 9 amino acid deletion isoform possessed notable structural differences in the regions between FN3 and immunoglobin (Ig) domain when compared to that of full-length Axl-L isoform (isoform 1) 16. This important finding reinforces our hypothesis that the deletion of these 9 amino acids may lead to functional and structural differences between *Axl-S* and *Axl-L* in humans.

Bioinformatics analysis of Axl pre-mRNA showed that a polypyrimidine sequence (CTCCTCTCTGTCCTTTCTTC) was present in the adjacent intron of *Axl* exon 10. This sequence is similar to the preferred binding sequence of PTBP1, suggesting that Axl exon 10 may be regulated by AS of PTBP1. Subsequently, by comparing sequence homology, we found that this polypyrimidine sequence is highly homologous among different species (i.e. mouse, rat, cow, horse, pig, chimpanzee, etc.), indicating that AS events of Axl exon 10 may exist in various species.

In this study, we found that the isoform of Axl, with exclusion of exon 10, was greatly increased in highly metastatic liver cancer cells. Isoform-specific knockdown of *Axl-S* inhibited cancer cell migration, invasion, and metastasis. Furthermore, the *Axl-S* isoform exhibited more robust binding with Gas6 than the *Axl-L* isoform and is more capable of activating downstream PI3K-AKT and ERK signaling pathways. Mechanism analysis showed that Axl exon 10 could be regulated by PTBP1. Further studies demonstrated that PTBP1 and *Axl-S* are required for the process of liver cancer cell invasion and metastasis. Overall, our results highlight the biological significance of the *Axl-S* isoform and PTBP1 in liver cancer invasion and metastasis. Our results also identified PTBP1 as an important splicing regulator that controls AS of Axl pre-mRNA, thereby promoting the invasion and metastasis of liver cancer cells.

## Materials and Methods

### Cell culture

293T, LO2 cells and human liver cancer cell lines (HepG2, SMMC7721, Bel7402, Huh-7, MHCC97H) were provided and identified by Guangzhou Institute of Biomedicine and Health. HCCLM3 were purchased from Keygene Biotechnology Company Limited. Liver cancer cell lines were cultured in 1640 (Gibco, Carlsbad, CA, USA). 293T cells were cultured in DMEM (Gibco, Carlsbad, CA, USA). All media was supplemented with 10% FBS (Gibco, Carlsbad, CA, USA), and 1% penicillin-streptomycin solution (BBI, China). All cells were cultured at 37 °C in an atmosphere containing 5% CO_2_.

### Plasmid construction

For construction of short hairpin RNA (shRNA) vectors, the shRNA primers (**Table S1**) of Axl-S, Axl-L, and Axl were designed. In addition, we draw a structural diagram to explain the design of Axl-L and Axl-S knockdown sites (**Figure S9**). Two forward primers and two reverse primers were denatured at 95 °C for 10 min. Subsequently, double-strand oligonucleotides were cloned into the sites *Hind* III and *Bgl* II of the pSuper-Retro. For construction of over-expression vectors, pMXs-flag plasmid was digested with *Pme* I, and the linearized pMXs-flag plasmid was homologously ligated with the Axl-L-ORF to construct a pMXs-Axl-L plasmid. For the construction of the pMXs-Axl-S plasmid, the pMXs-Axl-L plasmid was used as a template with the upstream and downstream primers specifically designed to delete exon 10. Following PCR amplification and *Dpn* I treatment, the reaction product was ligated using T_4_ DNA ligase kit (Takara, Japan), and transformed into DH5α competent cells after overnight culture on ampicillin-containing plates. Finally, the unattached clones were verified by nucleic acid sequencing. For construction of pcDNA3.1-Axl-minigene vectors, the Axl (Genbank, NC_000019.10) minigene genomic sequences spanning exons 9 to 11 (3852 nt total) were amplified using primers Axl-mini-HR-FP and Axl-mini-HR-RP. Next, this fragment was cloned into the *Xho* I and *EcoR* I sites of the pcDNA3.1 (+) vector by using homologous recombination kits (Trelief™ SoSoo Cloning Kit, Beijing, China). The PTBP1 over-expression plasmid, pMXs-PTBP1, and PTBP1 knockdown plasmid, pSuper-shPTBP1, were constructed and preserved by our laboratory. The primers used in this paper were designed by our group using the NCBI sequence and were synthesized by Guangzhou Qsingke Biological Company.

### Construction of mutant plasmid

For construction of the pcDNA3.1-Axl-minigene mutant plasmid, mutant primers Axl-in9-1-FP and Axl-in9-1-RP (or Axl-in9-2-FP and Axl-in9-2-RP) were designed and synthesized. PCR amplification was carried out using the pcDNA3.1-Axl-minigene plasmid as a template. Subsequently, 1 μL of *Dpn* I was added to 20 μL of the PCR product. After 1 h of reaction at 37 °C, 10 μL of the reaction product was transformed into DH5α cells. Positive clones were selected and the plasmid was extracted after verification by sequencing. Using the constructed pcDNA3.1-Axl-minigene-in9-1-Mu plasmid as a template, and Axl-in9-2-FP and Axl-in9-2-RP as primers, pcDNA3.1-Axl-minigene-in9-1+2-Mu was constructed according to the above procedure. For construction of PTBP1 RRMs deletion mutant plasmids, the pMXs-*Axl-L* plasmid constructed above was used as a template with the upstream and downstream primers specifically designed to delete the RRMs fragment.

### Transfection and Retrovirus infection

All the plasmids were transfected into cells and preparation of retrovirus viral particles as described in our previous work 17.

### Semi-quantitative reverse transcriptase PCR (RT-PCR) and quantitative real-time PCR (qPCR)

RT-PCR and qPCR are described in our previous work 17. Total RNA was extracted from cells using Trizol reagent (Invitrogen, USA) as described by the manufacturer. The RNA was then converted to cDNA via reverse transcriptase using a cDNA synthesis kit (TaKaRa, Japan) according to the manufacturer's instructions. For RT-PCR, 1 μL of the reverse transcription product was amplified by Ex Taq (TaKaRa, Japan) using primers pcDNA3.1-T7-FP, Axl-E9-FP, and Axl-E11-RP (**Table S1**). The inclusion and exclusion forms of exon10 of Axl were detected by RT-PCR. The positions of these two forms are depicted on 2% agarose gels. Image J software was then used for grayscale analysis by the ratio of exon skipping means the grayscale value of Axl-L/ Axl-S. For qPCR, synthesized cDNA was used in qPCR experiments with the SYBR Green mix (TaKaRa, Japan) and analyzed with a Bio-Rad Real-Time detection system (Bio-Rad, USA). The relative expression of target genes was calculated and normalized using the 2^-ΔΔCt^ method. The primers are shown in table S1.

### Western blot assay

Cell lysates were separated by 12% SDS-PAGE and transferred to the PVDF membrane. The membrane was then blocked with TBST buffer (TBS containing 0.1% Tween 20 and 5% non-fat dry milk) for 1 h, followed by an overnight incubation with primary antibody. AKT primary antibody (C67E7) and phosphorylated Ser473 AKT primary antibody (4060T) were purchased from Cell Signaling Technology Company. ERK primary antibody (AF1051), phosphorylated Thr202 ERK primary antibody (AF1891), and β-actin primary antibody (AF0003) were purchased from Beyotime Company. Axl primary antibody (AF8412) was purchased from Abbkine Company. Phosphorylated Tyr702 Axl primary antibody (AF8523) was purchased from Affinity Company. U2AF2 (4ab044957) and Gas6 primary antibody (4ab081330) were purchased from 4A Biotechnology Company. After being extensively washed, the membranes were incubated with HRP-labeled secondary antibodies at room temperature for 2 h. Finally, the membranes were washed 4 times with TBST and visualized by BeyoECL Moon kit (Beyotime, China), imaging was then performed using a biochemi-luminometer (General Electric, AI600). Grayscale quantifications of bands were performed using Image J software (National Institutes of Health, USA).

### Cell proliferation assay

5×10^3^ cells suspended in 100 μl medium were seeded into 96-well plates and incubated at 37 °C with 5% CO_2_ for 2 days. Cell proliferation was measured at day 2 by adding 10 μl CCK8 (Beyotime) into each well and incubating for 4 h at 37 °C. The OD_450_ values of each well were measured by microplate reader (Gen5, Ver2.1) and used to plot growth curves.

### Wound-healing assays

For wound-healing assays, cells were scratched onto the plate using a 100 μL pipette tip, and then cultured in 1640 medium. The wound size was measured at 0 and 48 h, respectively. For each sample, 6 areas were randomly selected and viewed at 10 × 10 magnification (scale bars: 100 μm). Quantification of wound-healing was performed using Image J. Scratch area width (W) was used to determine the extent of wound-healing, and cell migration ability was evaluated using “relative distance of wound closed.”

### Invasion assays

For invasion assays, cells were seeded in the upper chamber of the Transwell chambers with Matrigel (Corning, USA), 36 h later, cells in the upper chamber were fixed in 4% paraformaldehyde for 15 min and stained for 5 min in the dark, at room temperature, with 1 μg/mL 4', 6-diamidino-2-phenylindole (DAPI) (Beyotime, China). Cells were visualized under a fluorescent microscope (ZEISS, AXIOVERT A1). Six random fields were captured at 200× magnification. The invasive ability was calculated as the mean number of cells in all fields. The experiments were carried out three times independently.

### *In vivo* xenograft tumor formation and metastasis assay

Xenograft tumor formation assay was conducted as previously described 18. 4-week-old male nude mice (BALB/c-nu/nu) (*N*=6) were purchased from Beijing Vital River Laboratory Animal Technology Company Limited. The animals were maintained under Specific Pathogen Free (SPF) conditions and were provided food and water ad libitum. The mice were subcutaneously injected with indicated cells (2×10^6^ cells in 200 μl serum-free media) at each flank. For *In vivo* metastasis assay, indicated HCCLM3 cells (1×10^6^ cells in 200 μl serum-free media) were injected into the tail vein of 4-week-old BALB/C female nude mice (weighing 18-22 g, *N*=6). After 60 days of normal feeding and inhalation anesthesia with isoflurane, *in vivo* imaging of the mice was performed using an IVIS® Lumina III imager. The imaging mode was set to GFP fluorescence, and photon intensity was calculated for each mouse. Following sacrifice 60 days following HCCLM3 cell administration, lungs were harvested at necropsy and fixed in paraformaldehyde. Lungs were then embedded in paraffin for H&E (Haematoxylin-Eosin) staining to analyze the number of tumor nodules. All animal procedures were performed in accordance with the Jinan University Experimental Animal Care Commission.

### Immunohistochemistry (IHC)

For immunohistochemical analysis of ki-67, tissue sections were prepared as described previously 19. The primary antibody used was anti-ki-67 rabbit polyclonal antibody (1:250) (Servicebio, GB13030-2).

### MS2-GFP-Immunoprecipitation (MS2-GFP-IP)

The pcDNA3.1-Axl-minigene plasmid was digested with *Xho* I, and the linearized plasmid was homologously ligated with the 12×MS2 sequence to construct a pcDNA3.1-Axl-minigene-12×MS2 plasmid. Then, 10 μg of pMS2-GFP and pcDNA-Axl-minigene-MS2-12× plasmid was co-transfected into the corresponding HCCLM3 cells, and the nucleoprotein of liver cancer cells was extracted 48 h later. Subsequently, 3 mg of the cell nucleoprotein was pre-cleared with 50 μl protein G-agarose (Santa Cruz, American) at 4 °C for 2 h. The supernatant was then incubated with GFP antibodies with gentle shaking at 4 °C, overnight. This was followed by the addition of 50 μl of protein A/G-agarose for another 2 h. Lastly, the beads were washed and then re-suspended in 50 μl of 2×SDS-PAGE loading buffer and boiled for 10 min before Western blot detection.

### MS2-GFP-RNA-Immunoprecipitation (MS2-GFP-RIP)

10 μg of pMS2-GFP and pcDNA-Axl-minigene-MS2-12× plasmid were co-transfected into the corresponding HCCLM3 cells. Following 48 h, ultraviolet cross-linking was performed for 30 min. Next, the nuclear protein of the liver cancer cells was extracted and a mixture of 3 mg of cell nucleoprotein and GFP antibody-Protein G magnetic beads was combined in a vertical homogenizer for 4 °C overnight. This was followed by rinsing with a washing buffer for 10 min each, three times in total. Subsequently, 500 μl of Trizol was added to the magnetic bead complex and RNA was extracted and subjected to reverse transcription using a random primer before qPCR detection.

### Cross-link immunoprecipitation (CLIP)

CLIP was performed to verify the binding between PTBP1 and *Axl* pre-mRNA, which was performed as previously described 20. Briefly, RNA and protein complexes were cross-linked at 400 mJ/cm^2^ for 10 min (Stratagene). Immunoprecipitation was carried out with either anti-Flag antibody (Beyotime, 10 μg per reaction) or nonspecific IgG coupled with 50 μl protein G magnetic beads. Then, RNA was extracted from complexes and used to synthesize cDNA with random primers. Finally, RNA enrichment was measured by RT-PCR with primers specific for Axl mRNA.

### RNA pull-down assay

The RNA pull-down assay was carried out by synthesized biotinylated Axl RNA (Tsingke, China) as a probe. Using Pierce™ Magnetic RNA-Protein Pull-Down Kit (Thermo Scientific, USA) to perform the experiment. For each sample, 50 μl streptavidin magnetic beads were used to capture the biotin-labeled RNA. The products of RNA and nuclear protein complexes were washed and analyzed by Western blot.

### RNA-seq

The total RNA extraction was carried out as previously described 21. In brief, isolation of total RNA and RNC-RNA was accomplished using TRIzol Reagent, according to the manufacturer's instructions. Total RNA was subjected to RNA-seq. The polyA+ mRNA was then selected from the total RNA samples by RNA Purification Beads (Vazyme). The cDNA library products were generated using VAHTS mRNA-seq V2 Library Prep Kit for Illumina and sequenced using the Illumina HiSeq X Ten. Library construction and sequencing was performed at Shenzhen Chi-Biotech Corporation. High-quality reads were kept for the sequence analysis by the Illumina quality filters. The mRNA abundance was normalized using rpkM 22. Genes with > 10 mapped reads were considered quantified genes 23. The edgeR package method was adopted to analyze the differential expression genes 24.

### Bioinformatics analysis

Using UALCAN database (http://ualcan.path.uab.edu) 25 to analyze the expression of PTBP1 and Axl. Among 371 tumor samples of liver hepatocellular carcinoma dataset, 361 are hepatocellular carcinoma, 7 are hepatocholangiocarcinoma, 3 are fibrolamellar carcinoma. Using R2: Genomics Analysis and Visualization Platform (http://r2.amc.nl) to analyze clinical significance of PTBP1/ Axl.

### Statistical analysis

Results are expressed as the mean ± Standard Deviation (SD) with n representing the number of individual animals per experimental group. One-way (or two-way) analysis of variance (ANOVA) and Tukey's post-hoc analysis was used to evaluate statistical significance. Differences were considered statistically significant at P<0.05. All statistical analyses were performed using GraphPad Prism Version 5.0 software. Quantitative data are shown as mean and SD of at least three independent and separate experiments performed in triplicate.

## Results

### Oncogenic role of *Axl-S* isoform in human liver cancer cells

**Figure 1:**
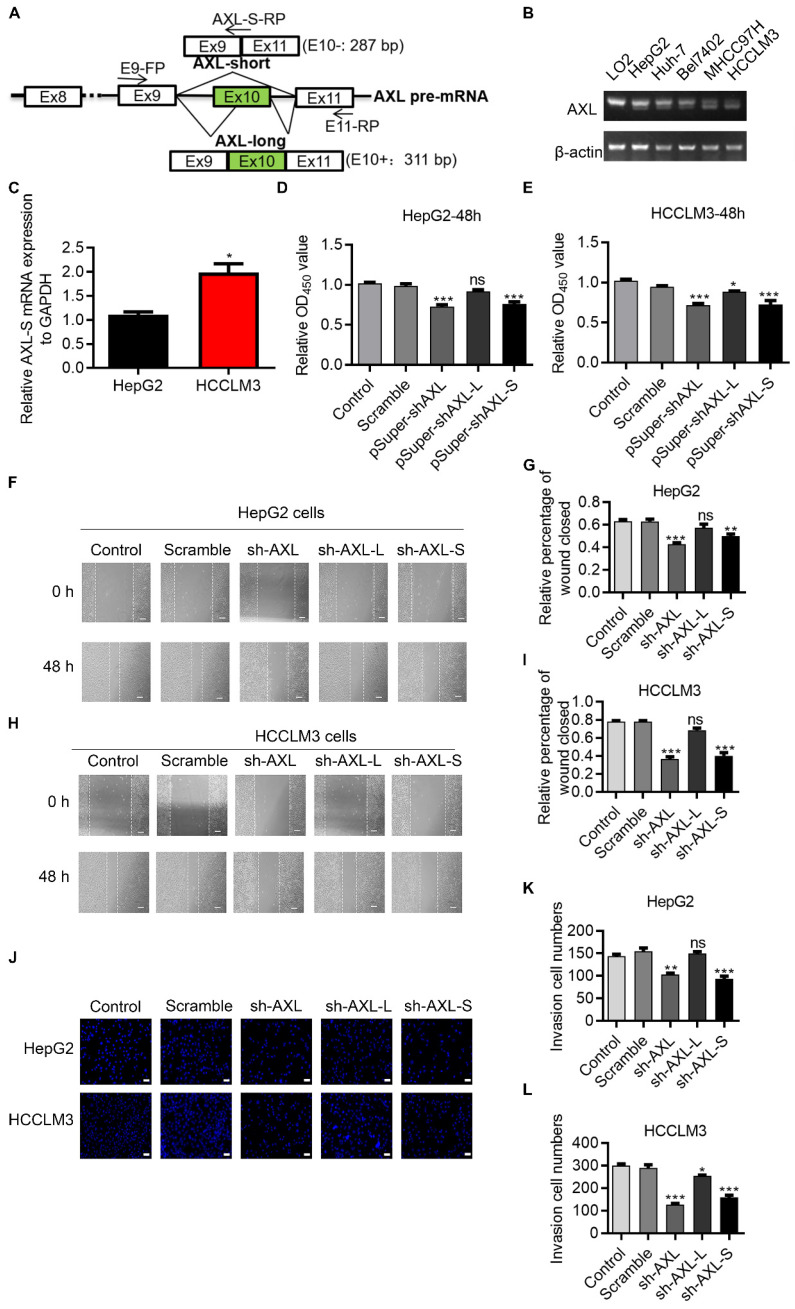
** Oncogenic role of *Axl-S* isoform in human liver cancer cells.** (A) Illustration of Axl pre-mRNA and Axl long and short isoforms; the arrow indicates the design site for the primer. Half of the *Axl-S*-RP sequence is located in exon 9 and half in exon 11. (B) RT-PCR was used to detect the difference in expression of Axl isoforms between HepG2 and HCCLM3. The primers used were Axl-E9-FP and Axl-E11-RP. The above panel shows the relative expression levels of Axl-S isoform in LO2, HepG2, Huh-7, Bel7402, MHCC97H and HCCLM3. (C) qPCR was used to detect the difference in expression of* Axl-S* isoform between HepG2 and HCCLM3. The primers used for qPCR detection of *Axl-S* isoform were Axl-E9-FP and Axl-S-RP. (D-E) The CCK8 assay was used to determine the 48h proliferation of HepG2 and HCCLM3 cells in different groups. The control levels were set to a value of 1. (F-G) Wound healing experiments were performed to examine the effects of knockdown of *Axl*, *Axl-L,* or *Axl-S* on the migration of HepG2 liver cancer cells. (H-I) Wound healing experiments were performed to examine the effects of knockdown of *Axl*, *Axl-L,* or *Axl-S* on the migration of HCCLM3 liver cancer cells. The magnification is 100× and the scale length is 100 μm. (J) The effect of *Axl*, *Axl-L,* or *Axl-S* knockdown on the invasive ability of HepG2 and HCCLM3 liver cancer cells, examined by Transwell assay. The magnification is 200× and the scale length is 50 μm. (K-L) One-way ANOVA and Tukey's post-hoc test were conducted to analyze the invasion HepG2 or HCCLM3. Data are presented as mean ± S.D. (*N*=3). The “*, **, ***” indicate “P<0.05, 0.01, 0.001” versus the control group, respectively.

Human *Axl* has 20 exons. Exon 10 on Human *Axl* is subject to AS regulation, which generates two transcript variants dependent on inclusion/exclusion of exon 10 (**Figure 1A**). We first investigated expression levels of *Axl* splice variants in normal liver cells (LO2), low metastatic potential HCC cell lines (HepG2, Bel7402 and Huh-7), high metastatic potential HCC cell lines (HCCLM3 and MHCC97H cells) 26, 27. We found that in high-metastatic HCCLM3 and MHCC97H liver cancer cells, the proportion of Axl-S isoforms was higher than that of normal liver cells LO2 and low-metastasis HepG2, Bel7402 and Huh-7 liver cancer cells (**Figure 1B**). The result qPCR also shows that in high-metastatic HCCLM3 liver cancer cells, the proportion of *Axl-S* isoforms was higher than that of low-metastasis HepG2 liver cancer cells (**Figure 1C**). We speculated that the ratio of the *Axl-S* isoform to total *Axl* variants may be related to the metastatic ability of liver cancer cells. Next, we analyzed whether Axl isoforms affect cell proliferation and metastasis using HepG2 and HCCLM3 human liver cancer cells. We designed an independent shRNA targeted against exon 10 for isoform-specific knockdown of *Axl-L*, and a shRNA to specifically knock down the *Axl-S* isoform, and then verified their efficiency (**Figure S1A-F**). We then assessed the effects of Axl isoform depletion on cell proliferation. Cell proliferation assays showed that knockdown of Axl and specific knockdown of *Axl-S* significantly inhibited the proliferation of HepG2 and HCCLM3 cells (**Figure 1D-E**). However, there were no significant effects in HepG2 cells (**Figure 1D**), and only weak inhibition in HCCLM3 cells when knocking down the *Axl-L* isoform (**Figure 1E**).

Axl has been reported to regulate cell invasion and migration 28-31. We posited that the two *Axl* isoforms caused by exon 10 inclusion or exclusion, might have different effects on cancer cell migration and invasion. To test this, we explored whether there was a difference in the effect of Axl isoforms on tumor metastasis through wound-healing and invasion assays. Wound-healing (**Figure 1F-I**) and invasion assays (**Figure 1J-L**) showed that knockdown of *Axl-S* significantly inhibited the migration and invasion of liver cancer cells, while knockdown of *Axl-L* had no significant effects (or only weak inhibition) on the invasion and migration of liver cancer cells. These data revealed that *Axl-S* and *Axl-L* had different effects on cell migration and invasion for HepG2 cells and, to a lesser degree, HCCLM3 cells. These data also indicate that the proportion of *Axl-S* isoform is correlated with tumor metastasis ability.

### *Axl-S* isoform has a more robust binding ability with the Gas6 ligand and is more capable of activating downstream pathways

**Figure 2:**
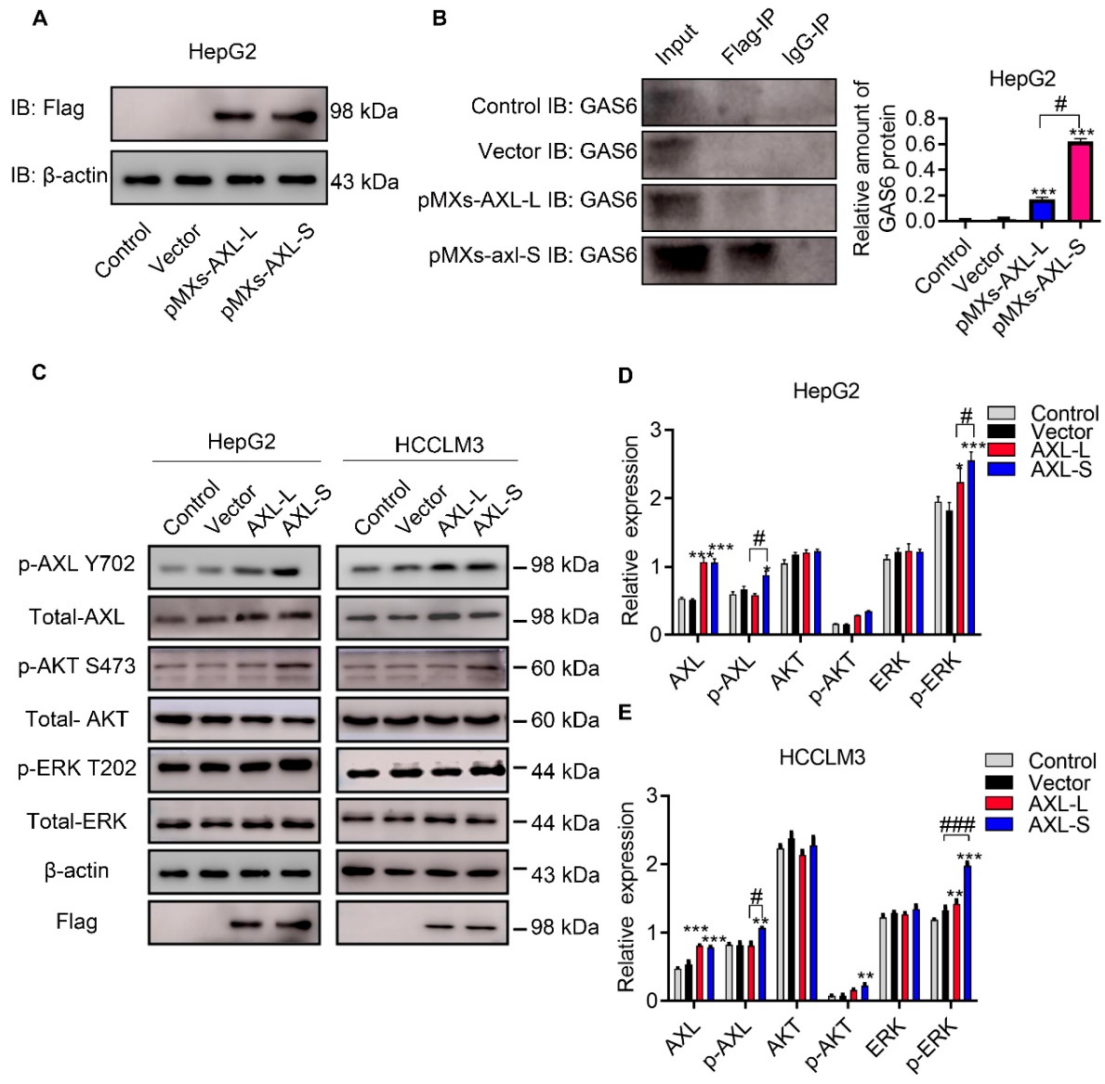
***Axl-S* isoform has a more robust binding ability to Gas6 ligands.** (A) Western-blot was used to detect the over-expression of *Axl-L* and *Axl-S* isoforms in HepG2 cells. (B) Left: Co-IP assay was used to detect the binding ability of *Axl-L* and *Axl-S* isoforms to Gas6 ligands; right: The relative amount of Gas6 protein bound in HepG2 cells over-expressing *Axl-L* or *Axl-S*. Data were analyzed using Student's T-test. The control levels were set to a value of 1. Data are presented as mean ± S.D. (*N*=3). The “***” indicates “P<0.001” versus the pMXs-*Axl-L* group. (C) The effect of *Axl-L* and *Axl-S* over-expression on Axl and downstream AKT and ERK signaling pathway proteins was detected by Western-blot. Statistical analysis of the effects of over-expression of Axl isoforms on AKT and ERK signaling pathway proteins in HepG2 cells (D) and HCCLM3 cells (E). The relative expression of p-Axl represents the relative expression of p-Axl to Axl. The relative expression of p-AKT represents the relative expression of p-AKT to total Axl. The relative expression of p-ERK represents the relative expression of p-ERK to total ERK. The relative expression of total Axl, AKT, ERK represents the relative expression of total Axl, AKT, ERK to β-actin. Two-way ANOVA and Tukey's post-hoc test was used to analyze the data. Data are presented as mean ± S.D. (*N*=3). The “*, **, ***” indicate “P<0.05, 0.01, 0.001” versus the control group, respectively. "#" represents P<0.05, "##" represents P<0.01, and "###" represents P<0.001. “#, ##, ###” indicates statistical difference between over-expressed *Axl-L* and *Axl-S* experimental groups.

The majority of Axl signaling occurs in a ligand-dependent manner mediated by Gas6 8. Clinically, Gas6 expression in tumor specimens has been identified as an adverse prognostic factor for several cancer types, including urothelial, ovarian, lung adenocarcinoma, gastric cancer, and glioblastoma 32. Together, these studies implicate Gas6/Axl signaling as an important pathway driving tumor growth and metastasis. Over the past decade, research has focused on elucidating the functional role of Gas6/Axl signaling in tumor progression. Whether the difference in function between *Axl-S* and *Axl-L* isoforms results from interaction with Gas6, is unclear. To test this, we first over-expressed *Axl-L* and *Axl-S* in HepG2 cells, respectively. Subsequently, the over-expression levels of *Axl-S* and *Axl-L* were tested using flag antibodies. Western-blot was then used to verify how much Gas6 was bound to each group. After confirming that levels of over-expressed *Axl-L* and *Axl-S* were similar, the difference in the binding level of Axl isoforms to Gas6 was verified using immunoprecipitation experiments (antibody: IP: Flag; IB: Gas6). The results showed that the *Axl-S* isoform had a more robust binding ability with Gas6 than *Axl-L* under the premise of similar over-expression efficiency (**Figure 2A-B**). Next, we verified whether there was a discrepancy in the activation of downstream pathways between *Axl-L* and *Axl-S* isoform. Western-blot results indicate that over-expression of *Axl-L* or *Axl-S* isoforms activates downstream ERK signaling pathways. However, in the case of similar expression levels, over-expression of *Axl-S* promotes phosphorylation of Axl and ERK to a greater degree than the *Axl-L* isoform (**Figure 2C-E**). These results indicate that the Axl-S isoform has a stronger binding affinity for Gas6 than the Axl-L isoform. Compared with Axl-L, Axl-S has a stronger effect on Axl phosphorylation and phosphorylation of downstream target proteins.

### *Axl* exon 10 is subject to the regulation by splicing factors PTBP1

**Figure 3:**
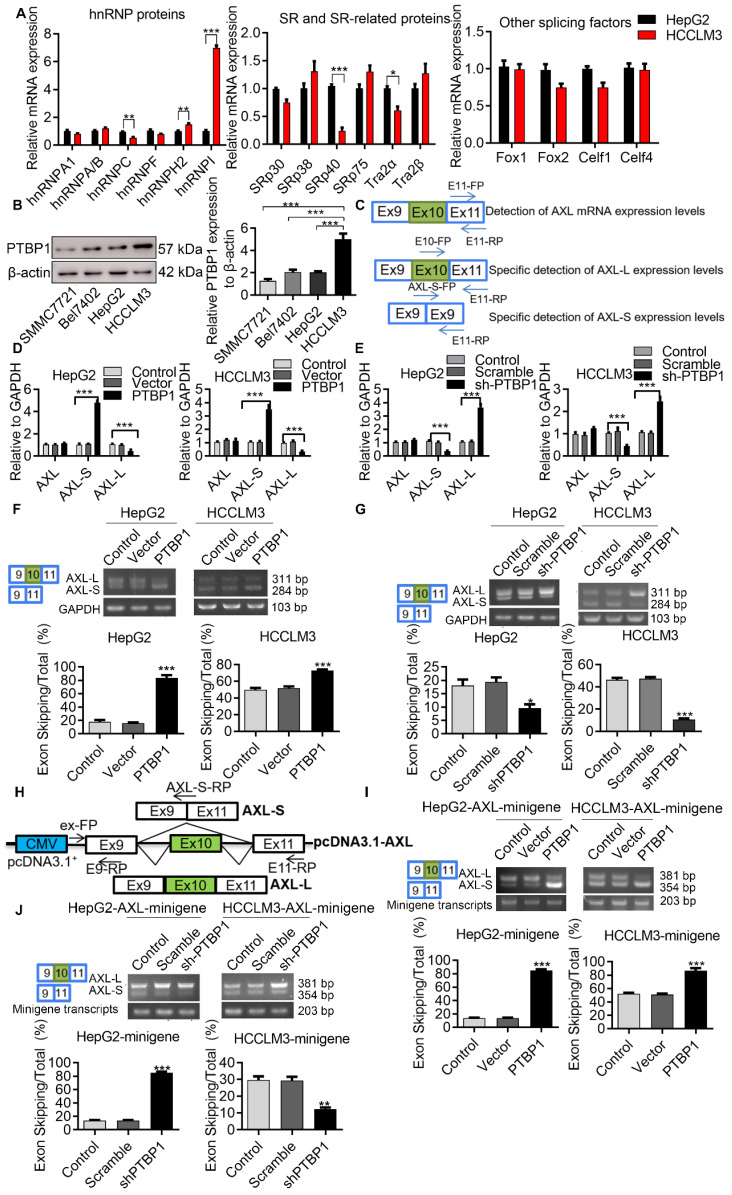
***Axl* exon 10 is subject to the regulation by splicing factors PTBP1.** (A) qPCR was used to detect differentially expressed hnRNP, SRs family proteins and other splicing factors in HCCLM3 cells and HepG2 cells. The control levels were set to a value of 1. (B) Western-blot was used to detect the differential expression of PTBP1 in four liver cancer cells. The control levels were set to a value of 1. (C) Graphical representation of qPCR primers for specific detection of *Axl* and *Axl-L*, *Axl-S* isoforms. Half of the *Axl-S*-FP sequence is located in exon 9 and half in exon 11. qPCR detection of over-expression (D) or knockdown (E) of *PTBP1* on the expression levels of *Axl* and* Axl-L*, *Axl-S* isoforms in HepG2 and HCCLM3 cells. The control levels were set to a value of 1. RT-PCR detection of over-expression (F) or knockdown (G) of *PTBP1* on the expression levels of *Axl-L* and* Axl-S* isoforms in HepG2 and HCCLM3 cells. (H) Graphical and specific primer design sites for pcDNA3.1-Axl-minigene. The primers for the internal reference of exogenous Axl-minigene were ex-FP and E9-RP, and the primers for RT-PCR detection of exogenous Axl isoforms were ex-FP and E11-RP; ex-FP and *Axl-S*-RP can be used to specifically detect the expression level of the *Axl-S* isoform. RT-PCR detection of over-expression (I) or knockdown (J) of *PTBP1* on the minigene levels of *Axl-L* and* Axl-S* isoforms in HepG2 and HCCLM3 cells. Data are presented as mean ± S.D. (*N*=3), one-way (B, F, G, I and J) or two-way (A and D) ANOVA followed by Tukey's test were performed for statistical analysis. The “*, **, ***” indicate “P<0.05, 0.01, 0.001” versus the HepG2 group (A) or HCCLM3 group (B), respectively. The “**, ***” indicate “P<0.01, 0.001” versus the control group (D, F, G, I and J), respectively.

Serine/arginine-rich proteins (SRs) and heterogeneous nuclear ribonucleoproteins (hnRNPs) are well-investigated splicing factors that have major roles in the regulation of cellular AS events. In order to identify proteins involved in the AS regulation of Axl pre-mRNA, we set out to test which members of these splicing factors modulate Axl exon 10 inclusion/exclusion in liver cancer cells. We first analyzed the differentially expressed splicing factors in liver cancer cells with both high and low metastatic potential using transcriptome sequencing. Gene expression comparison in HCCLM3 cells revealed that 1233 genes were down-regulated and 1369 genes were up-regulated in HepG2 cells (**Figure S2A**). We also wanted to identify the differentially expressed splicing factors in HepG2 cells and HCCLM3 cells. The result of transcriptome sequencing showed that the expression levels of splicing factors hnRNPA/B, hnRNPC, SRSF1, SRSF5, etc. in the hnRNPs family, SRs family, and other splicing genes, were increased in HCCLM3 cells compared to HepG2 cells (**Figure S2B-D**). Subsequently, qPCR results showed that expression of PTBP1 in HCCLM3 was significantly higher than HepG2. As shown in (**Figure 3A**), the PTBP1 mRNA level in HCCLM3s was 7.05-fold higher than HepG2. Earlier, we used RT-PCR to detect the expression levels of EIF4G2, PPP3CC, PRKDC, SPAG9, HTRA2, Axl after over-expression or knockdown of PTBP1 in HepG2 cells. Moreover, through bioinformatics analysis, it was found that Axl pre-mRNA has a sequence that may bind to PTBP1 (data not shown). Therefore, we studied the specific molecular mechanism of PTBP1 regulating Axl alternative splicing in this article Western-blot analysis showed that the expression level of PTBP1 in HCCLM3s was 2.47-fold higher than HepG2s (4.73-fold vs SMMC7721, 2.46-fold vs Bel7402) (**Figure 3B**). In addition, through analysis of PTBP1 expression in liver cancer patient database, the results of the UALCAN database show that in hepatocellular carcinoma (HCC), compared with normal (N = 50), the expression of PTBP1 increased in the primary tumor (N= 371), and the expression of Axl decreased significantly (**Figure S3A**). The expression of PTPB1 in the four stages of liver cancer development was also higher than that in normal tissues, but the expression of Axl in the four stages of liver cancer development was also lower than that in normal tissues (**Figure S3B**). The expression level of PTBP1 was positively correlated with the degree of liver cancer deterioration (**Figure S3C**). The results of the R2: Genomics Analysis and Visualization Platform illustrate that there is a negative correlation PTBP1 and Axl in Tumor Hepatocellular Carcinoma-Llovet-91-MAS5.0-u133p2 (**Figure S3D**).

In order to determine the role of PTBP1 in the exon 10 splicing of *Axl* pre-mRNA, we designed specific primers for the detection of *Axl* or the *Axl-L*/*Axl-S* isoforms (**Figure 3C**). After verification of over-expression and knockdown efficiency, over-expression and knockdown experiments were performed (**Figure S4A-F**). We found that exon 10 of Axl pre-mRNA can generate* Axl-L* or *Axl-S* transcripts after different AS patterns, and that the AS pattern of *Axl* pre-mRNA at exon 10 is exon-skipping. Firstly, we examined the mRNA levels of *Axl-L* or *Axl-S* by qPCR in liver cancer cell lines. As shown in (**Figure 3D**), over-expression of PTBP1 in liver cancer cells significantly increased the expression of *Axl-S* while decreasing the expression of *Axl-L*. The opposite result can be obtained by knocking down PTBP1 (**Figure 3E**). Over-expression or knockdown of PTBP1 in HepG2 cells did not affect the overall expression level of Axl (**Figure 3D-E**). Subsequently, we examined the transcriptional levels of *Axl-L* or *Axl-S* by reverse transcription PCR (RT-PCR).

The results in (**Figure 3F**) demonstrate that the percentage of exon 10 skipping was significantly increased in the PTBP1-over-expressed HepG2 cells, compared with non-treated HepG2 cells. Similar results were obtained from HCCLM3 cells. In addition, to test whether the absence of PTBP1 attenuated the percentage of *Axl-S*, we performed a knockdown of PTBP1 in liver cancer cells using shRNA. The results of RT-PCR showed that the expression of* Axl-S* isoform was sharply decreased in the shPTBP1 liver cancer cells compared with untreated cells (**Figure 3G**).

To further illustrate that PTBP1 is involved in the AS of *Axl pre-*mRNA, we constructed a minigene analysis. The genomic fragment corresponds to human *Axl*, the flanking Introns of exon 10 (380-nt upstream and 3163-nt downstream), and constitutive exons 9 and 10 (**Figure 3H**). Firstly, we examined whether exon 10 skipping in minigene was regulated by PTBP1. The results of RT-PCR in (**Figure 3I**) showed that exon 10 skipping in minigene was significantly increased in liver cancer cells over-expressing PTBP1 compared with non-treated cells. Consistent with expectations, we observed the opposite result in liver cancer cells knocked down by PTBP1 (**Figure 3J**). Subsequently, we examined the effect of over-expression or knockdown of PTBP1 on the expression of the *Axl-S* isoform by qPCR. Results indicated that over-expression of PTBP1 increased the mRNA levels of *Axl-S* (**Figure S5A**), while knockdown of PTBP1 inhibited the expression of *Axl-S* (**Figure S5B**). Combined with the above results, we concluded that expression of PTBP1 promoted the splicing of the *Axl-S* isoform in *Axl* pre-mRNA.

### PTBP1 enhances AS of *Axl* exon 10 through its interaction with upstream Intron 9 sequences

**Figure 4:**
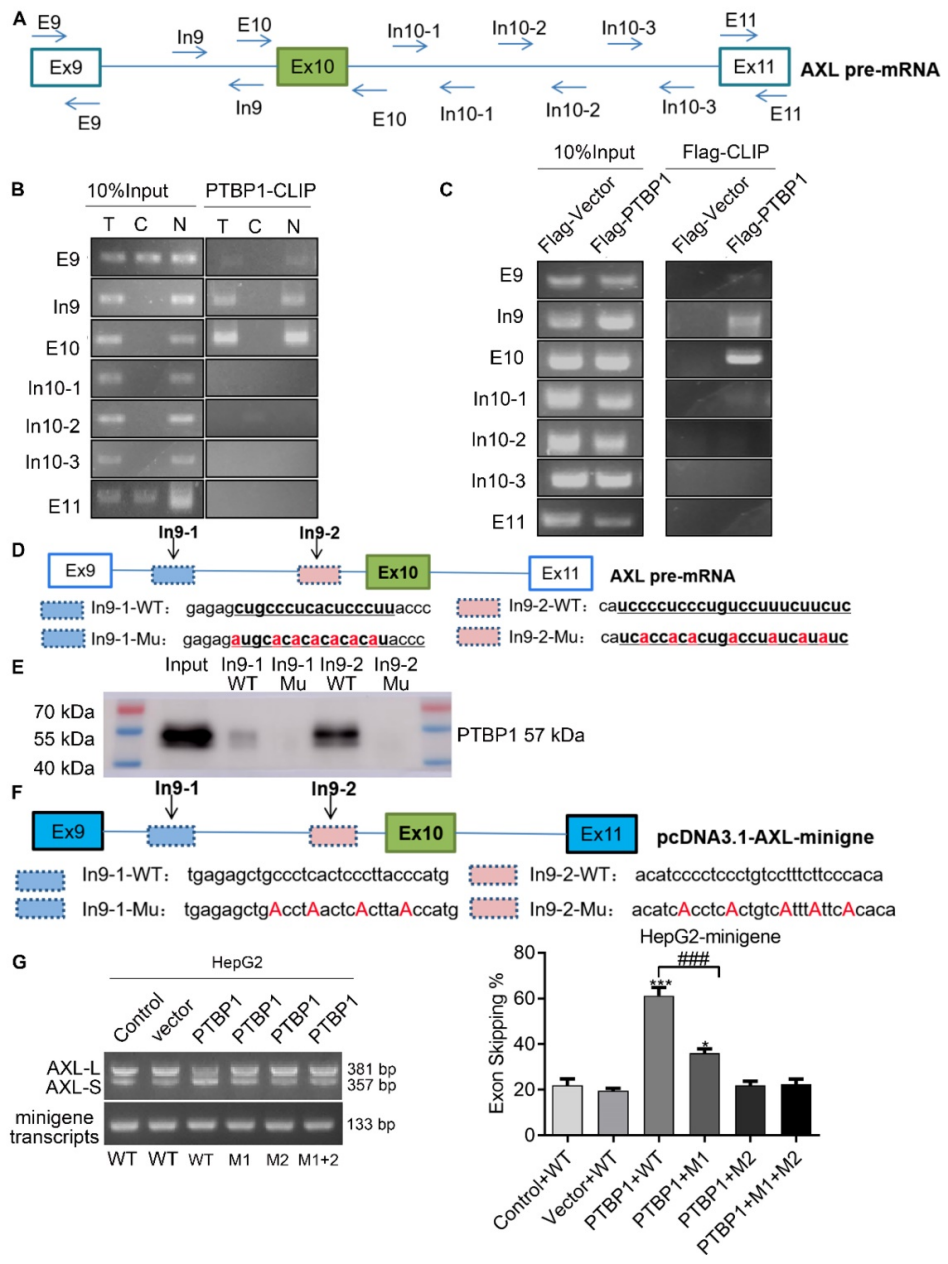
** PTBP1 enhances AS of *Axl* exon 10 through its interaction with upstream intron 9 sequences.** (A) The primer position used to detect the binding segment of PTBP1 and Axl pre-mRNA in the CLRIP experiment. The CLRIP assay was used to detect the binding segments of endogenous (B) and exogenous (C) PTBP1 to Axl pre-mRNA. "T" means Cytoplasmic and Nuclear lysate, "C" means Cytoplasmic lysate, "N" means Nuclear lysate. (D) The RNA fragment sequence used in the RNA pulldown assay and its position in the Axl pre-mRNA, the red font represents the mutated base. (E) RNA pulldown assay was used to detect the binding of PTBP1 to the biotinylated RNA fragments. (F) The mutated sequence of the Axl-minigene site-directed mutagenesis and its position in the Axl pre-mRNA, the red font is the mutated base. (G) RT-PCR was used to detect the effect of PTBP1 over-expression on the expression of *Axl-S* and Axl-L isoforms in different groups of HepG2 cells. Data are presented as mean ± S.D. (*N*=3), one-way ANOVA followed by Tukey's post-hoc test was performed for statistical analysis. The “*, ***” indicate “P<0.05, 0.001” versus the control group, respectively. The “###” indicates “P<0.001” versus the “PTBP1+WT” group.

Previous studies have reported that the consensus sequences for PTBP1 binding contain poly-pyrimidines, such as UCUU or CUCUCU. Using bioinformatics tools (Human Splicing Finder and RBP map), we obtained several potential PTBP1 binding sites on the upstream and downstream sequences of exon 10 in *Axl* pre-mRNA. The results suggested that PTBP1 might regulate splicing of *Axl* pre-mRNA. Next, we sought to examine the exact section for PTBP1 binding in *Axl* pre-mRNA *in vivo*. By looking up the *Axl* genome sequence, we identified several possible binding sequences located at exon 9, intron 9, exon 10, intron 10, and exon 11. We then designed primers in these sequences and performed cross-link immunoprecipitation (CLIP) to detect PTBP1 binding (**Figure 4A**). RT-PCR revealed that binding of PTBP1 to “In9” and “E10” sequences occurred most frequently (**Figure 4B**). In order to further determine the binding site of PTBP1 and *Axl* we transiently over-expressed PTBP1-Flag along with the Flag-vector in HepG2 cells as controls. The Flag antibody was used for CLIP experiments. RT-PCR results showed that binding of PTBP1 to “In9” and “E10” sequences also occurred more frequently than binding to the Flag-vector (**Figure 4C**). The above results indicate that the *Axl* pre-mRNA binding sequence for PTBP1 may be located at the “In9” and/or “E10” sequences.

We then narrowed the range of potential PTBP1 binding sequences via further bioinformatic analysis. We found one potential binding sequence **(CTGCCCTCACTCCCTT, In9-1)** for PTBP1 located 234 nt upstream of the 3' splice site of Intron 9, and another **(TCCTCTCTGTCCTTTCTTC, In9-2)** located 4 nt upstream of the 3' splice site of Intron 9 (**Figure 4D**). To determine the interactions between PTBP1 and the two potential binding sites, RNA-pulldown assays were performed using nuclear extracts from HepG2 cells. We synthesized 3'biotin-labled RNA oligomers, including the potential binding sequences and mutations of potential binding sites. The **CUGCCCUCACUCCCUU** (In9-1-WT) sequence in Intron 9 was mutated to **AUGCACACACACACAU** (In9-1-Mu). The **UCCUCUCUGUCCUUUCUUC** (In9-2-WT) sequence in Intron 9 was mutated to **UCACCACACUGACCUAUCAUA** (In9-2-Mu). RNA pulldown analysis showed that PTBP1 could bind with In9-1-WT and In9-2-WT, but not In9-1-Mu or In9-2-Mu. Interestingly, the binding of PTBP1 and In9-2-WT was more favorable than PTBP1 and In9-1-WT (**Figure 4E**). Furthermore, sequence alignment revealed that the UCCUCUCUGUCCUUUCUUC (In9-2-WT) sequence exists upstream of the variable exon 10 in several different mammalian species, but no similar sequences were found upstream of the constant exon 9 or exon 11. These results indicate that PTBP1 may bind to this conserved sequence (UCCUCUCUGUCCUUUCUUC) and subsequently regulate AS events of the Axl gene. To further verify whether the Axl pre-mRNA binding site for PTBP1 located in the above polypyrimidine sequence, we performed site-directed mutagenesis of the polypyrimidine sequence based on the original pcDNA3.1-Axl-minigene plasmid vector (**Figure 4F**). RT-PCR and qPCR results indicated that PTBP1 inhibited the AS process of wild-type Axl-minigene and increased expression of the *Axl-S* isoform (**Figure 4G and Figure S6**, **“PTBP1+WT” group**). However, PTBP1 significantly reduced the selective splicing regulation of Axl-minigene after single-segment mutation and double-segment mutation (**Figure 4 G and Figure S6, “PTBP1+M1”, “PTBP1+M2” and “PTBP1+M1+M2” group**). Interestingly, the inhibition of PTBP1's ability to regulate *Axl* AS was more pronounced after mutation of the "intron 9-2" sequence than the mutant "intron 9-1" sequence. The results of RNA pulldown and site-directed mutagenesis experiments indicated that the polypyrimidine sequence in the "intron 9-2" sequence is more important for PTBP1-mediated AS of Axl.

### PTBP1 inhibits AS of Axl exon 10 by competitively inhibiting U2AF2

**Figure 5:**
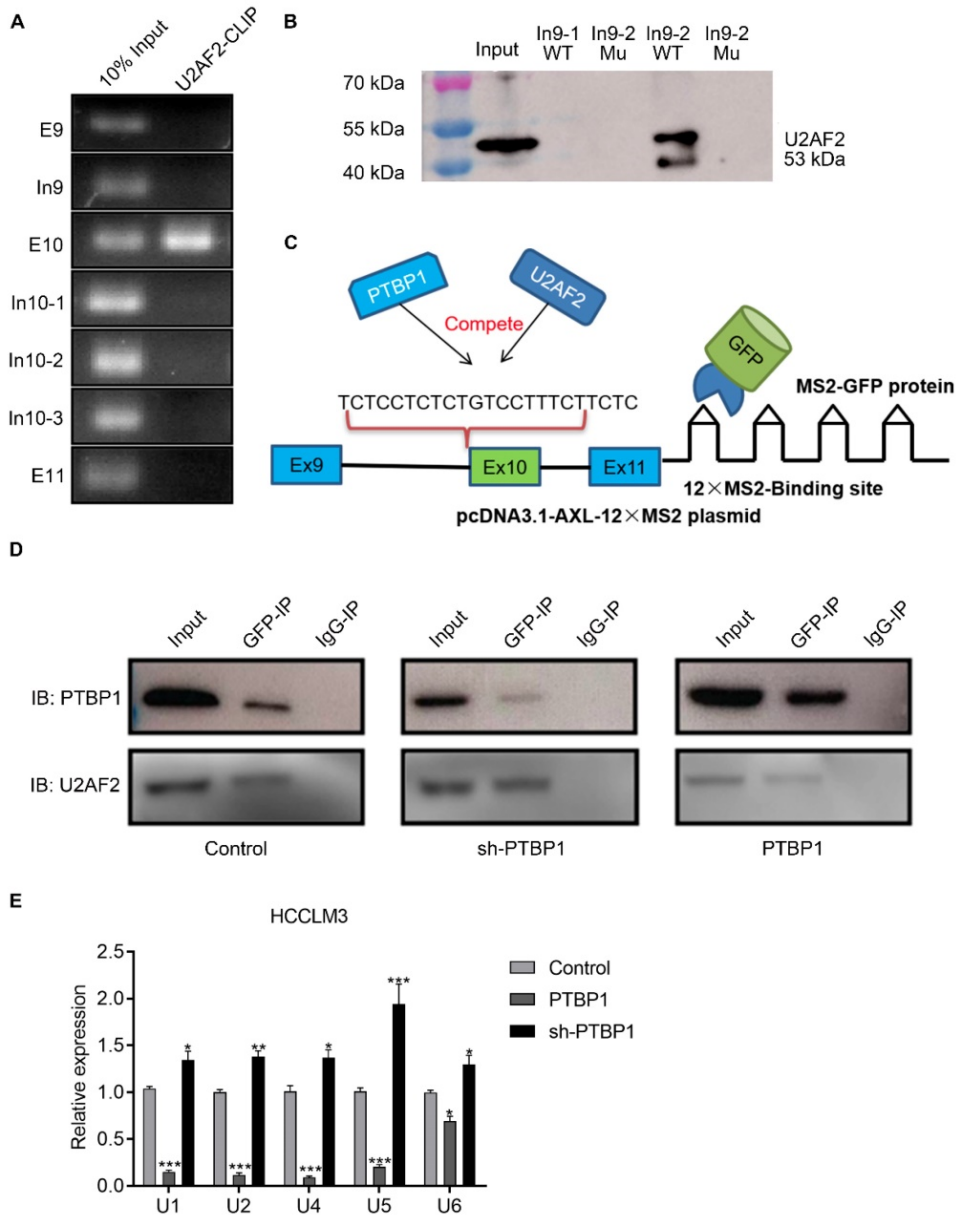
** PTBP1 inhibits AS of Axl exon 10 by competitively inhibiting U2AF2.** The U2AF2 binding sequence on Axl pre-mRNA was detected by CLRIP (A) and RNA pulldown assay (B). (C) Graphical representation of the MS2-GFP-IP system and its mode of operation. (D) The effect of PTBP1 over-expression or knockdown on the ability of U2AF2 to bind Axl pre-mRNA was investigated by the MS2-GFP-IP system. (E) The effect of PTBP1 on the ability of snRNAs to bind to Axl pre-mRNA was studied using the MS2-GFP-RIP system. The control levels were set to a value of 1. Data are presented as mean ± S.D. (*N*=3), two-way ANOVA followed by Tukey's test was performed for statistical analysis. The “*, **, ***” indicate “P<0.05, 0.01, 0.001” versus the control group, respectively.

During the AS process, U2AF2 is responsible for helping to recruit the U2 snRNP to the branch-point sequence, and the binding of U2 snRNP to the branch site allows the pre-mRNA to be successfully spliced. We hypothesized that PTBP1 acts through competition with U2AF2. CLIP and RNA-pulldown experiments showed that U2AF2 binds to the polypyrimidine sequence of the Axl intron 9-2 region, and its binding site is similar to PTBP1 (**Figure 5A-B**). We investigated whether PTBP1 and U2AF2 compete for binding to the polypyrimidine sequence, using the MS2-GFP system (**Figure 5C**). First, we transfected the psl-GFP and psl-Axl-12×MS2 plasmids into liver cancer cells, and then used GFP antibody for co-immunoprecipitation (specific recognition of MS2 sequences). Furthermore, Western-blot experiments were used to explore whether PTBP1 and U2AF2 competitively bind to Axl-minigene. We found that both PTBP1 and U2AF bind to Axl-minigene in liver cancer cells where PTBP1 is normally expressed. The degree of U2AF2 binding to Axl-minigene was significantly reduced in liver cancer cells knocked down by PTBP1. The opposite phenomenon was observed in liver cancer cells over-expressing PTBP1 (**Figure 5D**). snRNA (U1, U2, U4, U5, U6) is involved in the processing of RNA in the nucleus of eukaryotic cells. The snRNA binds to specific protein to become a small nuclear ribonucleoprotein (snRNP) involved in the splicing of the messenger RNA precursor (pre-mRNA). Using the 12×MS2 system, CLIP and qPCR experiments showed that over-expression of PTBP1 significantly reduced the binding of snRNA to Axl-minigene (**Figure 5E**). The above results indicate that PTBP1 competes with U2AF2 for binding to the polypyrimidine sequence on Axl-intron 9, thereby inhibiting the AS process of Axl exon 10. PTBP1 also inhibits AS of Axl by inhibiting the formation of a splicing complex.

### PTBP1 promotes the metastasis of liver cancer cells partially via regulating *Axl-S* levels

**Figure 6:**
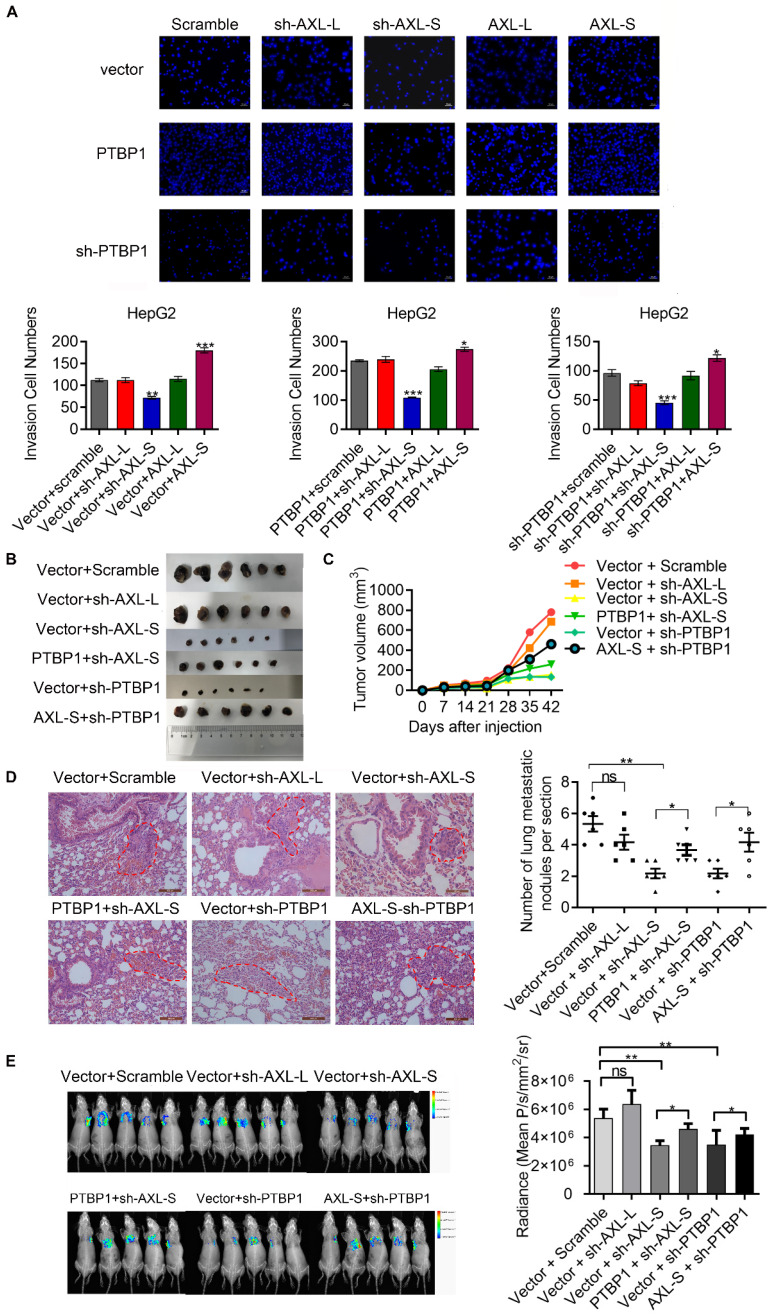
** PTBP1 promotes the metastasis of liver cancer cells partially via regulating *Axl-S* levels.** (A) The effect of PTBP1, Axl-L, and Axl-S expression levels on the invasive ability of HepG2 cells was examined by Transwell assay. The magnification is 200× and the scale length is 50 μm. Data are presented as mean ± S.D. (*N*=3), one-way ANOVA followed by Tukey's test was performed for statistical analysis. The “*, **, ***” indicate “P<0.05, 0.01, 0.001” versus the corresponding group, respectively. (B) Tumor size of different mouse groups. (C) The mean tumor volume of the tumors in the different groups of mice at 1, 2, 3, 4, 5, and 6 weeks. (D) HE staining was used to analyze the number of pulmonary metastases in different groups of nude mice. The magnification is 200× and the scale length is 100 μm. (E) In vivo imaging experiments in mice were performed to analyze the effects of PTBP1 and *Axl* isoforms on lung metastasis of liver cancer cells. Data are presented as mean ± S.D. (*N*=6), one-way ANOVA followed by Tukey's test was performed for statistical analysis. The “*, **, ***” indicate “P<0.05, 0.01, 0.001” versus the corresponding group, respectively.

Noting the upregulation of *Axl-S* in high metastatic liver cancer cells with over-expressed *PTBP1*, we wondered whether the effect of PTBP1 on migration and invasion of liver cancer cell lines is *Axl-S* dependent. After verification of the over-expression efficiency of *Axl-L* and *Axl-S* in liver cancer cells (**Figure S7A-D**), we conducted a two-factor experiment of *PTBP1* and *Axl* isoforms to investigate whether PTBP1 promotes liver cancer metastasis partially through regulating *Axl-S* levels. *In vitro* Transwell experiments showed that over-expression of *PTBP1* significantly increased the invasive ability of HepG2 cells, whereas knockdown of *Axl-S* significantly reversed this effect (**Figure 6A**). Similarly, over-expression of Axl-S significantly dampened the inhibitory effect of shPTBP1 on the invasive ability of HepG2 cells (**Figure 6A**). *In vivo* xenograft tumor formation experiments showed that knockdown of the *Axl-S* isoform significantly inhibits the proliferation (**Figure 6B-C, and Figure S8A-C**) and invasion (**Figure 6D**) of HCCLM3 cells, while knockdown of *Axl-L* has only a weak inhibitory effect. Interestingly, over-expression of *PTBP1* significantly reversed the effect of sh-*Axl-S* on proliferation inhibition of HCCLM3 cells (**Figure 6C**). Next, we studied whether PTBP1 regulates the expression level of *Axl-S* by *in vivo* animal imaging technology. *In vivo* imaging results showed that exogenous GFP-tagged HCCLM3 cells were specifically detected in the lung tissue of mice 60 days after injection of the HCCLM3 cells into the tail vein. It is worth noting that specific knockdown of *Axl-S* significantly inhibited the lung metastasis ability of exogenous HCCLM3 cells, whereas specific knockdown of *Axl-L* did not significantly inhibit the lung metastasis of HCCLM3 cells (**Figure 6E**). Similar to the in vitro results, *PTBP1* and *Axl-S* two-factor *in vivo* experiments showed that over-expression of PTBP1 reversed the inhibitory effect of *sh-Axl-S* on lung metastasis of HCCLM3 cells, and over-expression of *Axl-S* also reversed the inhibitory effect of sh-PTBP1 on lung metastasis of HCCLM3 cells (**Figure 6E**). In conclusion, through *in vitro* Transwell assays, *in vivo* xenograft, and *in vivo* imaging experiments, we concluded that PTBP1 partially promotes proliferation, invasion, and metastasis of hepatoma cells by regulating *Axl-S* levels.

## Discussion

Dysregulated AS events are commonly observed in liver cancer development and progression. For example, alterations in AS of the *GCH1*, *STK39,* and *TAFI* genes have been implicated in the context of multiple liver carcinogenesis mechanisms 33. In addition, upregulation of typical splicing proteins such as SRSF2, hnRNPL, hnRNPA2, hnRNPA2B1 and hnRNPAB were previously observed in liver cancers 33-37, although their misregulated splicing events have not been fully identified. In this study, our data showed that PTBP1 activates exclusion of Axl exon 10 to produce the *Axl-S* protein isoform. Both PTBP1 and *Axl-S* stimulate cell migration and invasion *in vitro* and promote tumorigenic and metastasis capacity of liver cancer cells *in vivo*. In this study, we have provided considerable evidence that PTBP1 directly regulates AS of Axl pre-mRNA.

The PTBP1 protein has four RRM domains. RBD1, RBD2, RBD3, and RBD4 bind the polypyrimidine sequence on the pre-mRNA either directly or through a ligand 38. Dr. Allain's studies showed that the pyrimidine sequences recognized by each RBD in PTBP1 are different. The sequences recognized by the RBD domain 1-4 are YCU, CU(N)N, YCUNN, YCN (Y stands for C or U, and N stands for any base) 39. However, there is still no clear conclusion regarding the characteristic sequences of PTBP1 binding. It is worth noting that there are a large number of pyrimidine-rich regions in the human genome. Since PTBP1 binds to the specific polypyrimidine sequence of pre-mRNA, it will affect AS of the target gene. Therefore, identifying the sequence in which PTBP1 binds to the target gene provides a theoretical basis for studying the molecular mechanism by which PTBP1 regulates AS. In this study, the binding sequence of PTBP1 to Axl pre-mRNA was examined by RNA-pulldown assay. Results showed that PTBP1 binds to the adjacent polypyrimidine sequence (**CUGCCCUCACUCCCUU** and **UCUCCUCUCUGUCCUUUCUUCUC**) of Axl exon 10. Subsequently, we designed a “pyrimidine-purine” mutant RNA fragment (**AUGCACACACACACAU** and **UCACCACACUGACCUAUCAUAUC**) based on the original RNA sequence. As expected, there was no binding of PTBP1 to the “pyrimidine-purine” (“T or C” mutated to “A”) mutant RNA fragment. These results corroborate those reported by Bielli et al 4. Their research showed that disruption of the binding site by replacement of two pyrimidines with two purines (**CCUUUCU** mutated to **CCGGUCU**) strongly affected PTBP1 binding to *BCL-X* RNA and impaired its ability to modulate *BCL-X* AS. The Bielli team also designed a “pyrimidine-pyrimidine” (“T” mutated to “C”) mutant RNA sequence (**CCUUUUUCUCCUUC** mutated to **CCUUCCUCUCCUUC**). Results from their study showed that the interaction between PTBP1 and “pyrimidine-pyrimidine” mutant RNA fragments still exist. The above studies indicate that PTBP1 can bind to different polypyrimidine sequences in the target gene pre-mRNA, thereby regulating AS of the target gene.

**Figure 7:**
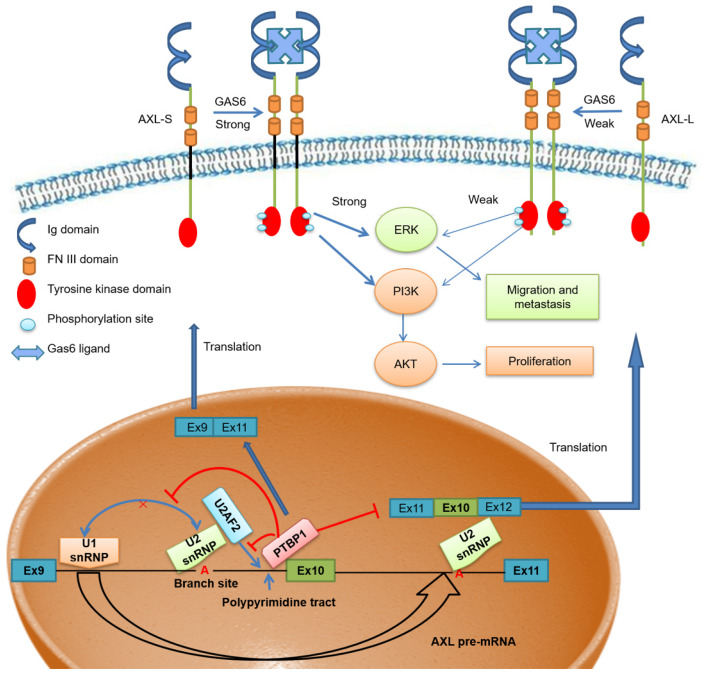
The effect of PTBP1-Axl signal axis on the metastasis process of liver cancer

Scientists have proposed multiple hypotheses for the molecular mechanism of PTBP1-mediated AS regulation. Studies have shown that PTBP1 binds to the polypyrimidine sequence adjacent to the variable exon and forms a circular structure in the corresponding region of the pre-mRNA, which can inhibit the binding of the splicing factor and the assembly of splicing complexes. As a result, the splicing process of the variable exon was inhibited 38, 40. Other studies have shown that PTBP1 specifically binds to the pyrimidine-rich sequence of the U1 snRNA stem-loop, rendering U1 snRNP unable to participate in the further assembly process of the splicing complex A, thereby also inhibiting the AS process of variable exons 41. However, there is no clear conclusion as to the molecular mechanism of PTBP1 regulating AS. Furthermore, a study by Pu Zhang et al. found that knockout of U2AF2 can promote the expression of *CD44s* (created by splicing out all variable exons), overexpressing U2AF2 can promote the expression of *CD44v* (generated by selectively including variant exon 6-14 )^42^. In addition, Jia Yi et al. have reported that knockout of U2AF2 can promote the expression of short isoform of the *KIAA0515* (NM 013318, exon 30); *TCERG1* (BI091338, exon 3); *BAT2D1* (AV650960, exon 6); *HNRNPH3* (NM 012207, exon 3); *UTRN* (NM 007124, exon 66) and so on 43. Based on these findings, we believe U2AF2 probably influences the alternative splicing of Axl. Our study demonstrates that PTBP1 binds to the adjacent polypyrimidine sequence of Axl exon 10 and competitively inhibits U2AF2 binding, thereby inhibiting the assembly of splicing complex A. We also showed that skipping Axl exon 10 generates the *Axl-S* isoform, which ultimately promotes the metastasis process of liver cancer (**Figure 7**). Based on these findings, we believe PTBP1 affects the assembly of splicing complexes in many ways, effectively inhibiting the splicing of variable exon 10 and promoting the production of the short isoform. In this study, the effect of PTBP1 on the splicing complexes during the AS of Axl and whether U2AF2 influences the alternative splicing of Axl independently was not well defined. In subsequent studies, on one hand, *in vitro* splicing and splicing complex assembly experiments should be used to study the effects of PTBP1 on the assembly of splicing complexes E, A, B, C1, and C; on the other hand, we should study the effect of U2AF2 alternation on the expression of *Axl-S* and *Axl-L*.

It is worth noting that Axl is not the only gene regulated by PTBP1. At present, PTBP1 has been found to be involved in the AS regulation of approximately 1500 genes 44. In different cell lines PTBP1 may regulate the selective splicing of other target genes and play a variety of functions. Interestingly, studies have shown that AS of individual genes may be regulated by a variety of splicing proteins. Therefore, the AS of Axl may also be regulated by other splicing factors. In the process of AS, the splicing factors are not in a one-to-one correspondence with the same variable exon of target genes, and the actual regulatory patterns may be diverse. For example, the AS of the apoptosis-related gene *BCL-X* is regulated by the splicing factors SRSF1 and PTBP1, which competitively bind to characteristic sequences on variable regions, promoting AS of *BCL-X* pre-mRNA in different ways, resulting in two opposite isoforms 4. Studies have also shown that the splicing factors SRSF1 and hnRNPA1 can mutually influence the selection of splice sites, thereby regulating the AS of downstream target genes 45. We screened the potential target genes of PTBP1 by RT-PCR, and the results showed that Axl is the main target gene of PTBP1 regulation in liver cancer cells. However, IP-MS technology should be used to identify other candidate splicing proteins involved in the Axl pre-mRNA AS process. Such technology could also be used to elucidate the molecular mechanisms by which multiple splicing proteins collectively regulate Axl exon 10 AS.

Related research results show that Axl is associated with metastasis in lung, breast, prostate, pancreatic, ovarian, colon and hepatocellular cancers and melanomas and gliomas 46-52. In addition, Gas6 / Axl system linked to multiple types of human cancer 53. Studies show that Axl-mediated exogenous Gas6 has an impact on migration 54, 55. Signaling pathways activated downstream of Axl/Gas6 include PI3K-AKT, MAPK-ERK, as well as several others. From the experimental results (**Figure 2**), we found that *Axl-S* has a stronger interaction with the Gas6 ligand under the same conditions compared to the *Axl-L* isoform. In the case of similar levels of over-expression, over-expression of *Axl-S* increased the level of phosphorylated Axl, thereby activating downstream PI3K-AKT and MAPK-ERK signaling pathways to a greater extent. The 3D structural analysis of *Axl-L* and *Axl-S* isoforms by Jong's research team showed significant structural differences in the regions between FN3 and Ig domain 16. Generally, biological function of a specific signaling pathway could be changed depending on interactions between the receptor and its ligand, as well as their domain folding structure. Our study also found that *Axl-S* and *Axl-L* have distinct functions for downstream pathway proteins, and that this difference is closely related to their structure. However, the work by Jong et al. was only based on *in silico* homology, and our speculation similarly lacks a structure-activity relationship study. Therefore, further experimental crystal structure studies would be necessary to explain these Axl AS effects.

In summary, our findings reveal tumorigenic roles for PTBP1 and the *Axl-S* isoform of Axl in liver cancer cell migration, invasion, and metastasis. We also identify PTBP1 as a key splicing regulator in controlling Axl pre-mRNA splicing, which plays an important role in mediating tumorigenesis of liver cancer cells. In-depth study of the PTBP1-Axl AS signal axis can provide new ideas for elucidating the metastasis mechanisms of liver cancer.

## Supplementary Material

Supplementary figures and table.
