# Supplementary material for: Skipping of exon 10 in Axl pre-mRNA regulated by PTBP1 mediates invasion and metastasis process of liver cancer cells
Source: Theranostics. 2020 Apr 27;10(13):5719–35. doi: 10.7150/thno.42010 (PMC7255001; doi:10.7150/thno.42010)
Supplement: Supplementary file 1 — Supplementary figures and table. [file thnov10p5719s1.pdf]

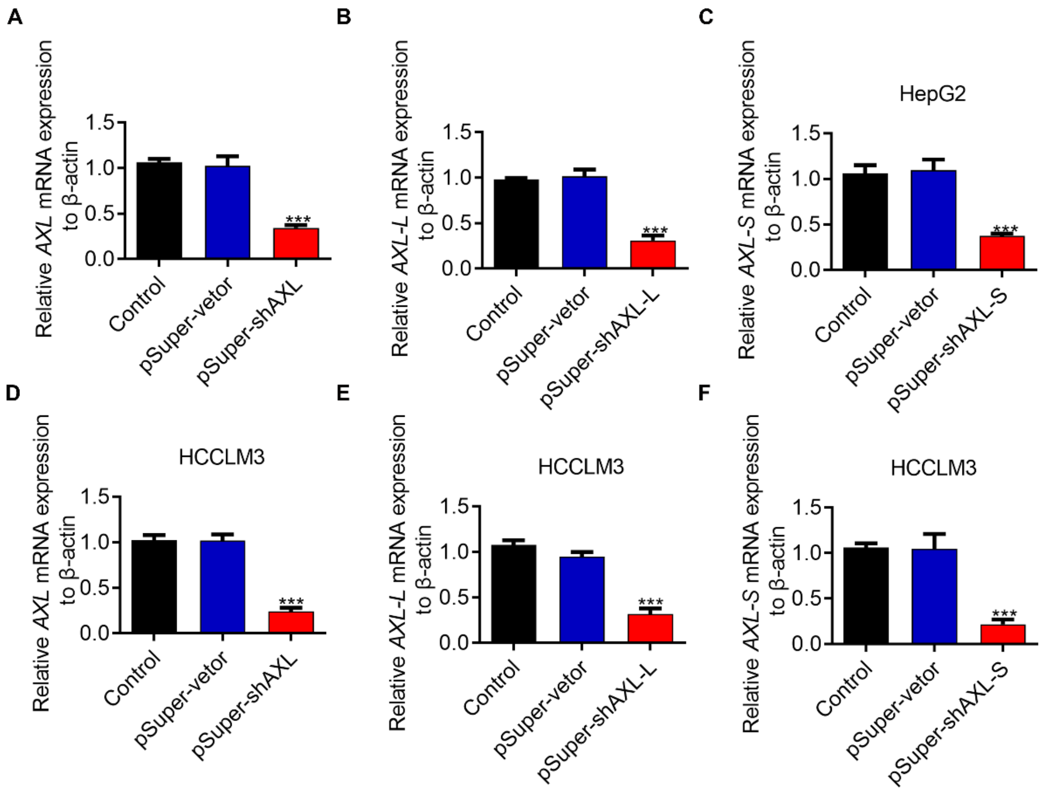

Figure S1. qPCR detection of knockdown efficiency of *Axl*, *Axl-S* and *Axl-L* in liver cancer cells

qPCR was used to detect the knockdown efficiency of *Axl* (A), *Axl-L* (B) and *Axl-S* (C) in HepG2 cells; qPCR was used to detect the knockdown efficiency of *Axl* (D), *Axl-L* (E) and *Axl-S* (F) in HCCLM3 cells. Data are presented as mean ± S.D. (*N*=3), and one way ANOVA followed by Tukey's test was performed for statistical analysis. The "***" indicates "P<0.001" versus the control group.

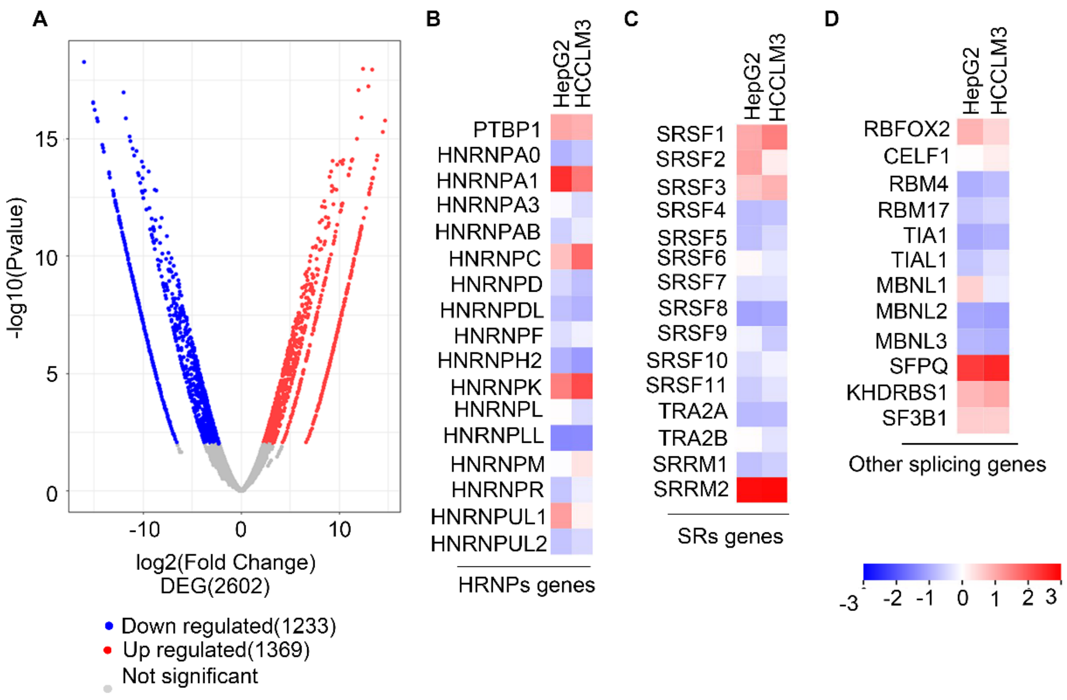

Figure S2. Transcriptome sequencing analysis of differentially expressed genes in high and low metastatic liver cancer cells

(A) Volcano Mapping Analysis of Differentially Expressed Genes in HCCLM3 and HepG2 Cells. Transcriptome sequencing analysis of differentially expressed hnRNP family proteins (A), SR family proteins (B) and other splicing proteins (C) in HCCLM3 cells and HepG2 cells.

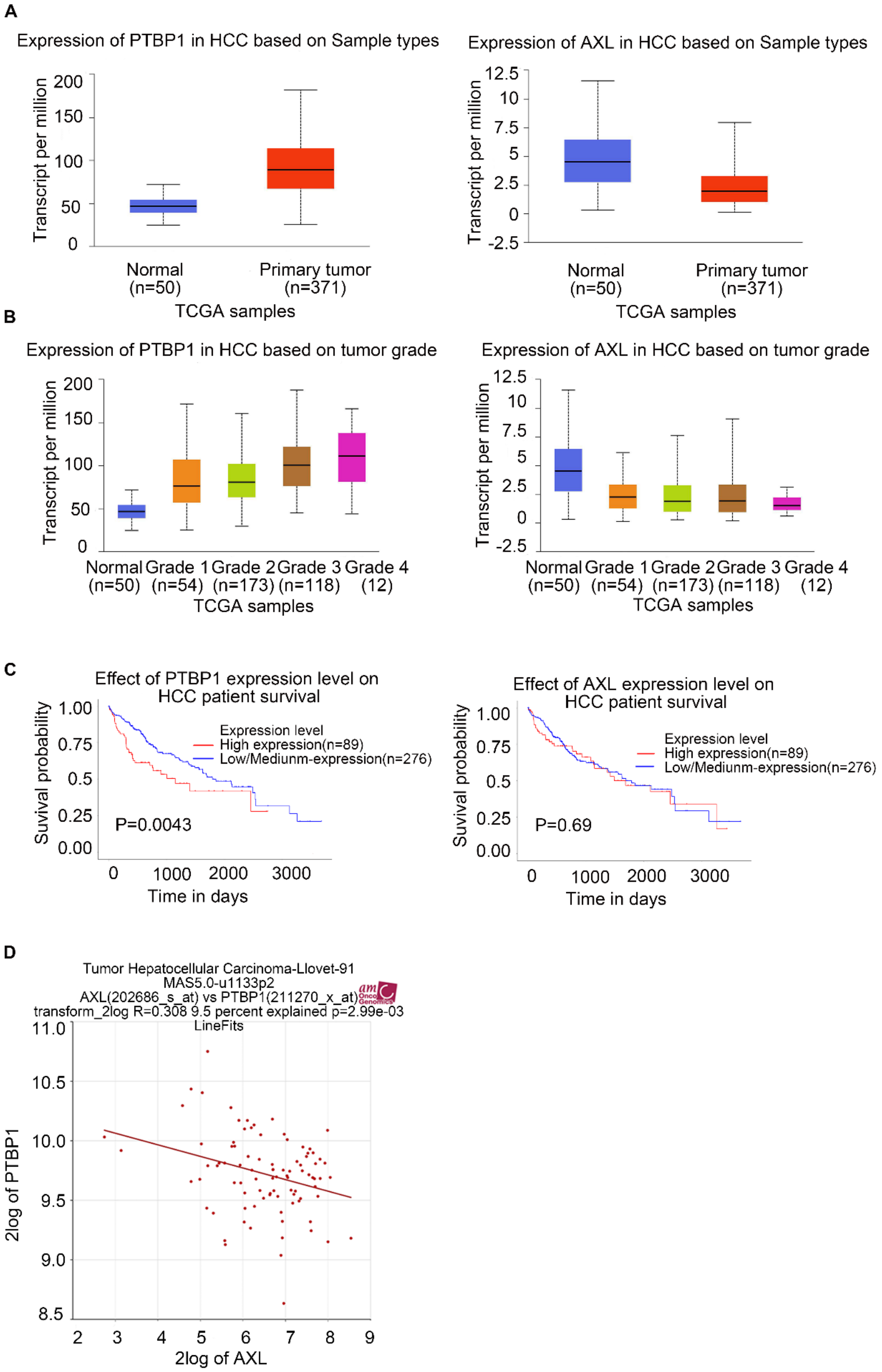

Figure S3. Clinical analysis of the effect of PTBP1 and Axl expression level on liver cancer

(A) The expression level of PTBP1 and Axl in liver cancer tissues and normal liver tissues. (B) The expression level of PTBP1 and Axl in different stages of liver cancer. (C) Effect of difference in expression level of PTBP1 and Axl on survival probability of patients with liver cancer. (D) The clinical significance of PTBP1/ Axl.

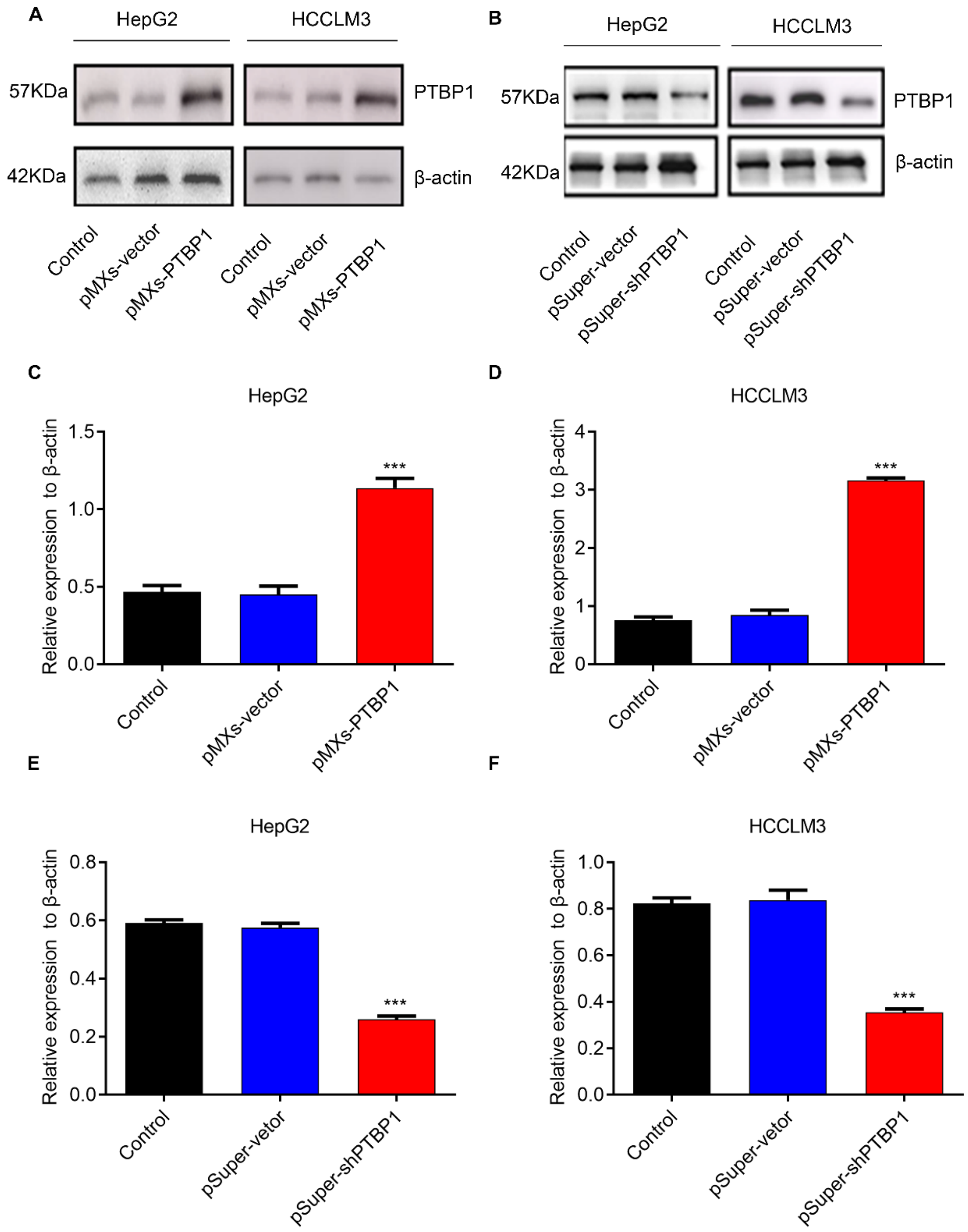

Figure S4. Verification of over-expression or knockdown efficiency of PTBP1

Western-blot detects the over-expression (A) or knockdown (B) efficiency of PTBP1. (C-D) Statistical difference analysis of PTBP1 over-expression efficiency; (E-F) Statistical difference analysis of PTBP1 knockdown efficiency. Data are presented as mean ± S.D. (N=3), one way ANOVA followed by Tukey's test was performed for statistical analysis. The “***” indicates “P<0.001” versus the control group.

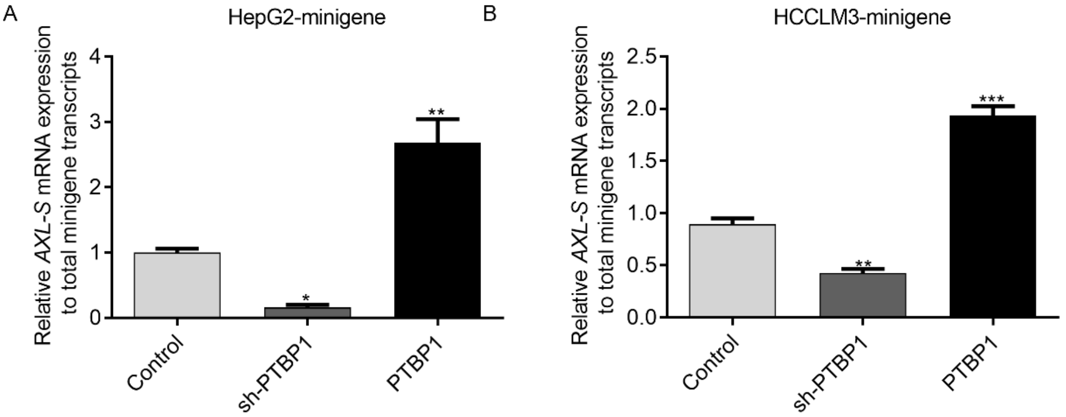

Figure S5. qPCR was used to detect the effect of PTBP1 on the *Axl-S* isoform (minigene level)

Over-expression or knockdown of the effect of PTBP1 on the expression levels of *Axl-S* isoform (minigene levels) in HepG2 (A) or HCCLM3 (B) cells. Data are presented as mean ± S.D. (N=3), one way ANOVA followed by Tukey's test was performed for statistical analysis. The “*, **, ***” indicates “P<0.05, 0.01, 0.001” versus the control group, respectively.

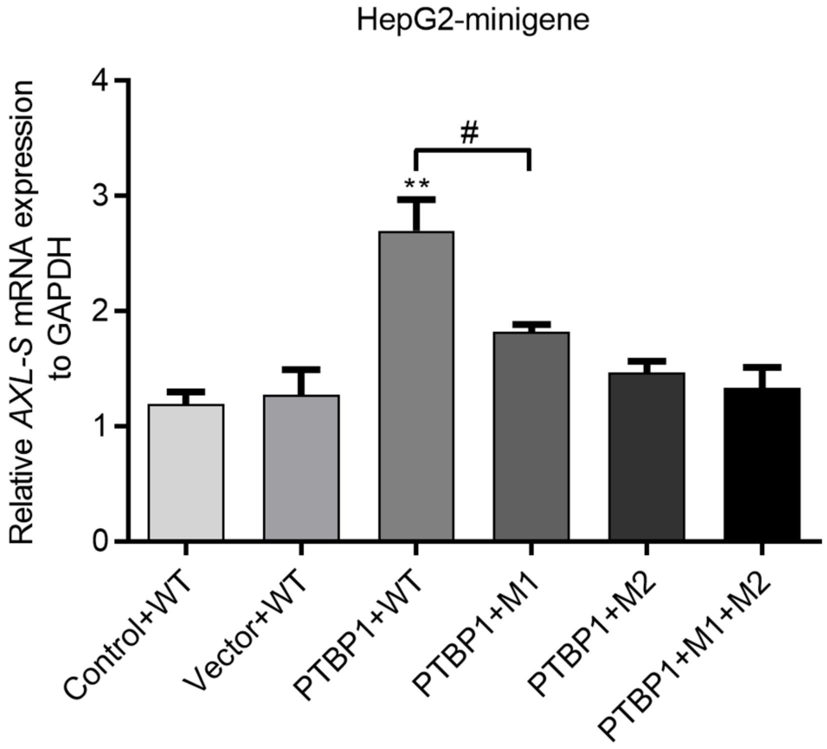

Figure S6. qPCR was used to examine the effect of PTBP1 on wild-type or mutant Axl-minigene

Data are presented as mean ± S.D. (*N*=3), one way ANOVA followed by Tukey's test was performed for statistical analysis. The "**" indicates "P<0.01" versus the control group; The "#" indicates "P<0.05" versus the "PTBP1+WT" group.

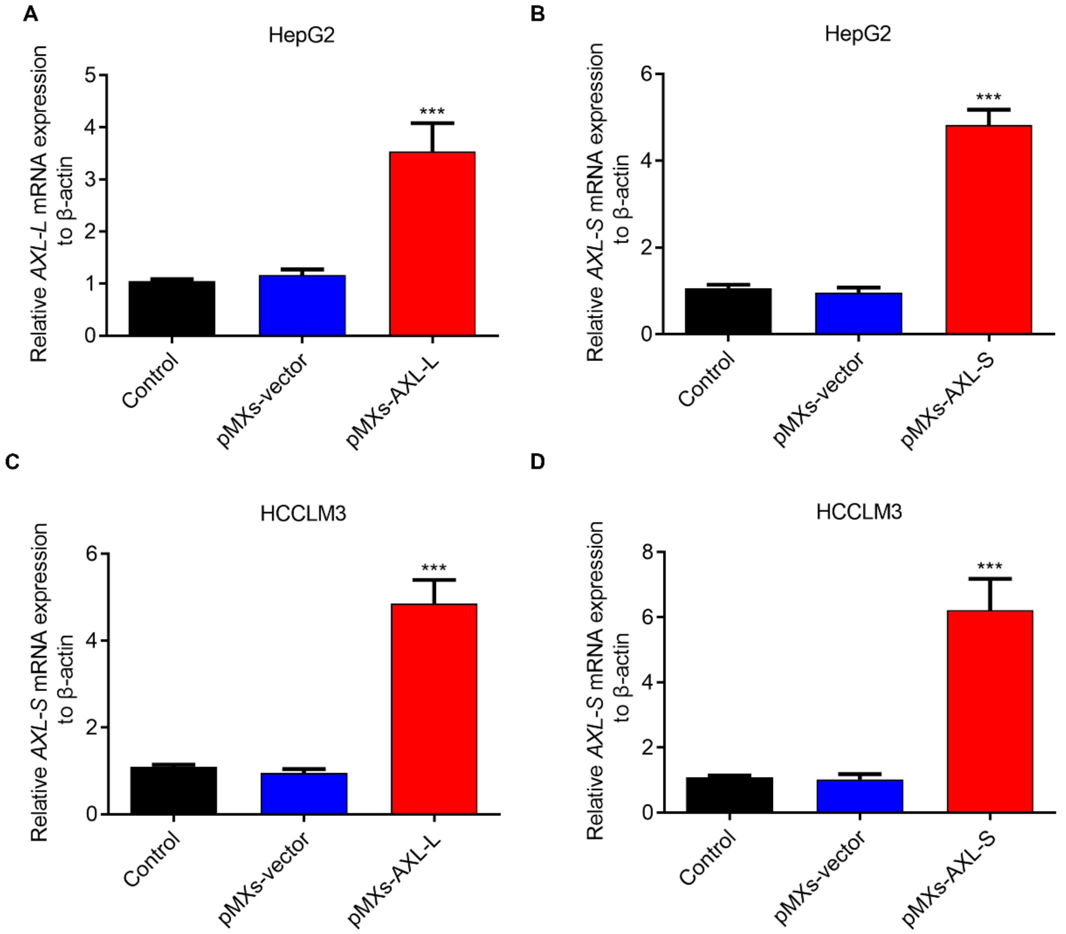

Figure S7. qPCR detection of over-expression efficiency of *Axl-L* or *Axl-S* isoforms in liver cancer cells

qPCR was used to detect the over-expression efficiency of *Axl-L* (A) and *Axl-S* (B) in HepG2 cells; qPCR was used to detect the over-expression efficiency of *Axl-L* (C) and *Axl-S* (D) in HCCLM3 cells. Data are presented as mean ± S.D. (*N*=3), and one way ANOVA followed by Tukey's test was performed for statistical analysis. The "***" indicates "$P<0.001$" versus the control group.

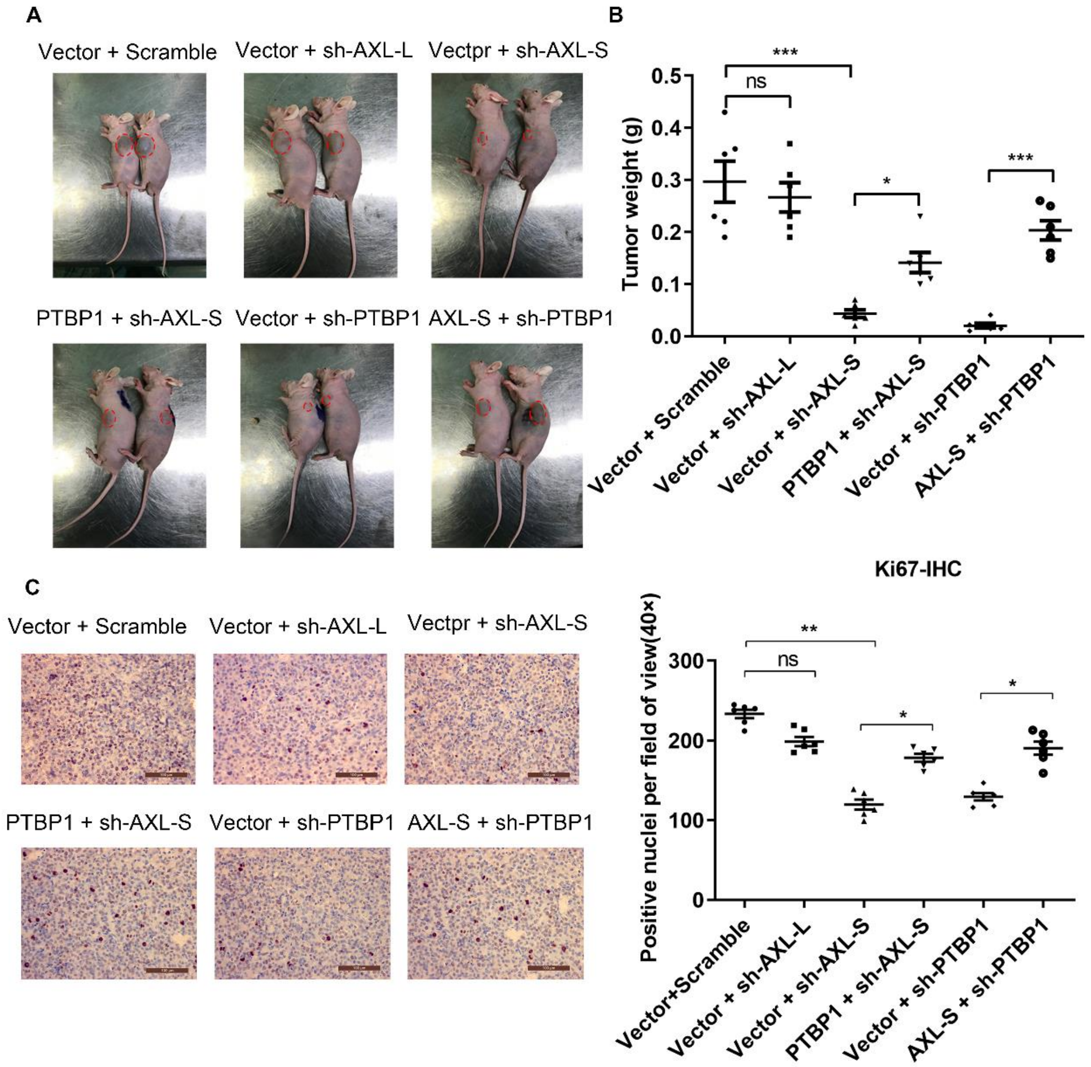

Figure S8. Effects of expression levels of PTBP1 and Axl isoforms on growth and proliferation of hepatoma cells

(A) Tumor size appearance of each group of mice, and the red circle marks the tumor. (B) The average weight of tumors in each group of mice. (C) The expression level of Ki67 in tumor tissues was analyzed by immunohistochemistry, and the average Ki67 positive cells in tumor tissues of each group were analyzed by image J software. Data are presented as mean ± S.D. (*N*=6), and one way ANOVA followed by Tukey's test was performed for statistical analysis. The "*, **, ***" indicates "P<0.05, 0.01, 0.001" versus the corresponding group, respectively.

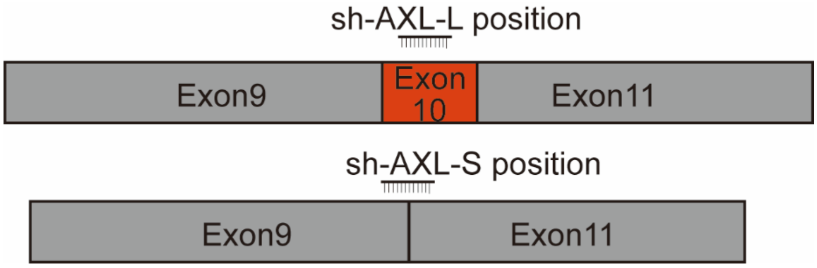

Figure S9. Structural diagram to explain the design of Axl-L and Axl-S knockdown sites.

Supplementary Table 1 List of primers

**Primer names**	**Sequences**	**Applications**
Axl-E9-FP	5'GTGCTAATGGACATAGGGCTAAG3'	Fig. 1 B, 1 C, 3 F, 4 B, 4 C and 5 A
Axl-E11-RP	5'CCATAACGGGTCTCCTTCTTTC3'	Fig. 1 B, Fig. 3 D, 3 E, 3 G, 4 B, 4 C, 5 A and Fig. S1 and S6
Axl-S-RT-RP	5'GGTTCCTTCACTGGGCGCC3'	Fig. 1 C and Fig. S5, S6
GAPDH-RT-FP	5'GACACCCACTCCTCCACCTTT3'	Fig. 1 C, Fig. 3 A, 3 D, 3 E, 3 F, Fig. S1 and S7
GAPDH-RT-RP	5'TGTTGCTGTAGCCAAATTCGTT3'	Fig. 1 C, Fig. 3 A, 3 D, 3 E, 3 F, Fig. S1 and S7
hnRNPA1-RT-FP	5'CTTTGCCTTTGTAACCTTTGACG3'	Fig. 3 A
hnRNPA1-RT-RP	5'TCTTTGGCTGGATGAAGCACTAG3'	Fig. 3 A
hnRNPA/B-RT-FP	5'TAGACCAGAAGGAGCACAGGC3'	Fig. 3 A
hnRNPA/B-RT-RP	5'TGGCTTCAGGATTCAGACCC3''	Fig. 3 A
hnRNPC-RT-FP	5'TAGTGCCCTCGAAACGTCAG3'	Fig. 3 A
hnRNPC-RT-RP	5'TAGTGCCCTCGAAACGTCAG3'	Fig. 3 A
hnRNPF-RT-FP	5'CAGGCAGAGTGGTGAGGCT3'	Fig. 3 A
hnRNPF-RT-RP	5'CTCGGTTCTGTGGGACTTGA3'	Fig. 3 A
hnRNPH2-RT-FP	5'GCTGGGTTTGAAAGGATGAG3'	Fig. 3 A
hnRNPH2-RT-RP	5'TGTGGTGCTCTGGAAACTGG3'	Fig. 3 A

PTBP1-RT-FP	5'TGGATGGTGCTCCTTCTCGT3'	Fig. 3 A
PTBP1-RT-RP	5'GCTGCTTCCTCGGTTGCTAG3'	Fig. 3 A
SRp30-RT-FP	5'GGGATGTCTGTTATGCTGATGTGC3'	Fig. 3 A
SRp30-RT-RP	5'GGGAACCCCTGCTTTGGTAT3"	Fig. 3 A
SRp38-RT-FP	5'CACTTGATTTCTACACTCGCCG3'	Fig. 3 A
SRp38-RT-RP	5'TACACATTCCTCCCTTCCTTGG3'	Fig. 3 A
SRp40-RT-FP	5'GGACGATACTCTGACCGTTTTA3'	Fig. 3 A
SRp40-RT-RP	5'CTTCCCCAGCTTGTCTCATG3'	Fig. 3 A
SRp75-RT-FP	5'TGTGGTGAGCGAGTAATTGTTG3'	Fig. 3 A
SRp75-RT-RP	5'TTCATTTTTGCGTCCCTTGTG3'	Fig. 3 A
Tra2α-RT-FP	5'AATCCAGATCCCAACACTTGCC3'	Fig. 3 A
Tra2α-RT-RP	5'ATCCACCCGAATTCTTCTACCA3'	Fig. 3 A
Fox1-RT-FP	5'AAAACAAGTCTCAGCCCAAGCG3'	Fig. 3 A
Fox1-RT-RP	5'CGAAAGTTACGAAACCAAATCCCT3'	Fig. 3 A
Fox2-RT-FP	5'TTGCCAAACTGCCCAAACAT3'	Fig. 3 A
Fox2-RT-RP	5'GCAGAGCAGCAACTCACCCA3'	Fig. 3 A
Celf1-RT-FP	5'AGATGTTTGTGGGCCAGGTT3'	Fig. 3 A
Celf1-RT-RP	5'TGGGAGGACTTTCATGTTGTGA3'	Fig. 3 A
Celf4-RT-FP	5'AAACGGACAGGCTGACAACG3'	Fig. 3 A
Celf4-RT-RP	5'CCTTCAGAACCGTAAGCTCGTA3'	Fig. 3 A
Axl-E11-FP	5'TGAAGGAACCTTCAACTCCTGCC3'	Fig. 3 D, 3 E, 4 B, 4 C and Fig. S1 A and S1 D.
Axl-E10-FP	5'TCCCTTACTCGTGCCACACCCTCAC3'	Fig. 3 D, 3 E, 4 B, 4 C, 5 A and Fig. S1 B, S1 E, S7 A and S7 C.

Axl-*S*-FP	5'GGCGCCCAGTGAAGGAACC3'	Fig. 3 D, 3 E and Fig. S1 C, S1 F, S7 B and S7 D.
Axl-*S*-RP	5'GGTTCCTTCACTGGGCGCC3'	Fig. 1 C, S5 and S6
pcDNA3.1-ex-FP	5'TAATACGACTCACTATAGGGAGACC3'	Fig. 3 H, 3 I and S5
Axl-E9-RP	5'CTGGGCGCCAGGCCTCCAGG3'	Fig. 3 H, 3I, 4 B, 4 C, 5 A and S5
Axl-*L*-ORF-HR-FP	5'**GTTAATTAAGGATCCGTTT<u>GCCACC</u>**ATGGCGTGGCGGTGCCCCAG3'	Construction of pMXs-*AXL*-*L*
AXL-*L*-ORF-HR-RP	5'**CGTCCTTGTAGTCTTGTTT**TCAGGCACCATCCTCCTGCCCT3'	Construction of pMXs-*AXL*-*L*
pMXs-*Axl*-*S*-FP	5'TGAAGGAACCTTCAACTCCTGC 3’	Construction of pMXs-*AXL*-*S*
pMXs-*Axl*-*S*-RP	5'CTGGGCGCCAGGCCTCCAG 3’	Construction of pMXs-*AXL*-*S*
pMXs-FP	5' GACCCCGGGGGTGGACCATCCTCT 3'	Sequencing
pMXs-RP	5' TTATCGTCGACCACTGTGCTG3'	Sequencing
sh*Axl*-1st	5'gatccccCGTGGAGAACAGCGAGATTTAttcaagaga3'	Construction of pSUPER-sh*AXL*
sh*Axl*-2nd	5'TAAATCTCGCTGTTCTCCACGtttta3'	Construction of pSUPER-sh*AXL*
sh*Axl*-3rd	5'TAAATCTCGCTGTTCTCCACGggg3'	Construction of pSUPER-sh*AXL*
sh*Axl*-4th	5'agcttaaaaaCGTGGAGAACAGCGAGATTTAtctcttgaa3'	Construction of pSUPER-sh*AXL*
sh*Axl*-*L*-1st	5'gatccccGGCAAGCACAGCCAGTCCACCttcaagaga3'	Construction of pSUPER-sh*AXL*-*L*
sh*Axl*-*L*-2nd	5'GGTGGACTGGCTGTGCTTGCCtttta3'	Construction of pSUPER-sh*AXL*-*L*

sh*Axl*-*L*-3rd	5'GGTGGACTGGCTGTGCTTGCCggg3'	Construction of pSUPER-sh*AXL*-*L*
sh*Axl*-*L*-4th	5'agcttaaaaaGGCAAGCACAGCCAGTCCACCtctcttgaa3'	Construction of pSUPER-sh*AXL*-*L*
sh*Axl*-*S*-1st	5'gatccccTGGCGCCCAGTGAAGGAACCttcaagaga3'	Construction of pSUPER-sh*AXL*-*S*
sh*Axl*-*S*-2nd	5' GGTTCCTTCACTGGGCGCCAtttttа3'	Construction of pSUPER-sh*AXL*-*S*
sh*Axl*-*S*-3rd	5' GGTTCCTTCACTGGGCGCCAggg3'	Construction of pSUPER-sh*AXL*-*S*
sh*Axl*-*S*-4th	5'agcttaaaaaTGGCGCCCAGTGAAGGAACCtctcttgaa3'	Construction of pSUPER-sh*AXL*-*S*
pSUPER (+19)	5' CGACGGTATCGATAAGCTTAAAAA 3'	Sequencing
pSUPER (-20)	5' TGTGTAGCGCCAAGTGCCCA 3'	Sequencing
Axl-mini-FP	5'GTGCTAATGGACATAGGGCTAA3'	Construction of pcDNA3.1-AXL-minigene
Axl-mini-RP	5'CCATAACGGGTCTCCTTCTTTC3'	Construction of pcDNA3.1-AXL-minigene
Axl-mini-HR-FP	5'**TAGTCCAGTGTGGTG**GAATTCGTGCTAATGGACATAGGGCTAA3'	Construction of pcDNA3.1-AXL-minigene
Axl-mini-HR-RP	5'**AACGGGCCCTCTAGA**CTCGAGCCATAACGGGTCTCCTTCTTTC3'	Construction of pcDNA3.1-AXL-minigene
Axl-Intron-9-1-MT-FP	5'TGAGAGCTGACCTAACTCACTTAACCATG3’	Construction of mutant pcDNA3.1-AXL-minigene

Axl-Intron-9-1-MT-RP	5'CATGGTTAAGTGAGTTAGGTCAGCTCTCA3'	Construction of mutant pcDNA3.1-AXL-minigene
Axl-Intron-9-2-MT-FP	5'ACATCACCTCACTGTCATTTATTCACACA3'	Construction of mutant pcDNA3.1-AXL-minigene
Axl-Intron-9-2-MT-RP	5'TGTGTGAATAAATGACAGTGAGGTGATGT3'	Construction of mutant pcDNA3.1-AXL-minigene
Axl-minigene-MS2-FP	5'**GCACAGTGGCGGCCG**CTCGAGCCGGGCCCTATATATGGA3'	Construction of pcDNA3.1-AXL-minigene-MS2 plasmid
Axl-minigene-MS2-RP	5'**AACGGGCCCTCTAGA**CTCGAGTCGATCGCGCGCAGATCT3'	Construction of pcDNA3.1-AXL-minigene-MS2 plasmid
Axl-In9-FP	5'TCTCTCCCTGACAGCCCTGACT3'	CLIP. Fig. 4 B, 4C and 5A
Axl-In9-RP	5'AAGGGAGTGAGGGTGTGGCACG3'	CLIP. Fig. 4 B, 4C and 5A
Axl-E10-RP	5'GAGAAACATGAAAGGGATGTGC3'	CLIP. Fig. 4 B, 4C and 5A
Axl-In10-1-FP	5'TCCTCCTCACATCCATCCCTCTC3'	CLIP. Fig. 4 B, 4C and 5A
Axl-In10-1-RP	5'GAATGAGAAGTGTGTTGTGCAATGG3'	CLIP. Fig. 4 B, 4C and 5A
Axl-In10-2-FP	5'CTCCATTCCACCCCTGCCCTTT3'	CLIP. Fig. 4 B, 4C and 5A
Axl-In10-2-RP	5'AGGGGCATGGTAAGCATATACAG3'	CLIP. Fig. 4 B, 4C and 5A
Axl-In10-3-FP	5'TCTCTCCCTCCCATTCCACACTC3'	CLIP. Fig. 4 B, 4C and 5A
Axl-In10-3-RP	5'AAGAGAGTGCCAGGATAGTTCA	CLIP. Fig. 4 B, 4C and

	GG3'	5A

Bold of *Axl-L*-ORF-HR-FP/RP are the homologous sequences of pMXs-Flag vector; **Bold** of Axl-mini-HR-FP/RP and Axl-minigene-MS2-FP/RP are the homologous sequences of pcDNA3.1-(+) vector; The underlined GCCACC is the Kozak sequence (Applied to pMXs-*Axl-L* and pMXs-*Axl-S* plasmids); The Double underline "T" or "A" represents the mutation site (Applied to Construction of mutant pcDNA3.1-Axl-minigene); The wavy line marks GAATTC and CTCGAG are the cleavage sites of *Eco*R Iand *Xho* I, respectively.
